# Effects of Two Chinese Herbal Formulae for the Treatment of Moderate to Severe Stable Chronic Obstructive Pulmonary Disease: A Multicenter, Double-Blind, Randomized Controlled Trial

**DOI:** 10.1371/journal.pone.0103168

**Published:** 2014-08-13

**Authors:** Genfa Wang, Baojun Liu, Yuxue Cao, Yijie Du, Hongying Zhang, Qingli Luo, Bei Li, Jinfeng Wu, Yubao Lv, Jing Sun, Hualiang Jin, Kai Wei, Zhengxiao Zhao, Lingwen Kong, Xianmei Zhou, Qing Miao, Gang Wang, Qingwei Zhou, Jingcheng Dong

**Affiliations:** 1 Department of integrated traditional Chinese and western medicine, Huashan Hospital, Fudan University, Shanghai, PR China; 2 Institute of integrated traditional Chinese and western medicine of Fudan University, Shanghai, PR China; 3 Respiratory Department, Jiangsu Province Hospital of TCM, Nanjing University of TCM, Nanjing, PR China; 4 Respiratory Department, Xiyuan Hospital CACMS, Beijing, PR China; 5 Respiratory Department, West China Hospital, Sichuan University, Chengdu, PR China; 6 Respiratory Department, the First Hospital Affiliated to Henan University of TCM, Zhengzhou, PR China; University of Texas Health Science Center at Tyler, United States of America

## Abstract

**Objective:**

The study aims to evaluate the efficacy and safety of two Chinese herbal formulae for the treatment of stable COPD.

**Methods:**

A multicenter, double-blind, double-dummy, and randomized controlled trial (RCT) was conducted. All groups were treated with additional conventional medicines. There were a 6-month treatment and a 12-month follow-up for 5 times. Primary outcomes included lung function test, exacerbation frequency, score of SGRQ. Second outcomes consisted of 6MWD, BODE index, psychological field score, inflammatory factors and cortisol.

**Results:**

A total of 331 patients were randomly divided into two active treatment groups (Bushen Yiqi (BY) granule group, n = 109; Bushen Fangchuan (BF) tablet group, n = 109) and a placebo group (n = 113). Finally 262 patients completed the study. BY granule & BF tablet increased the values of VC, FEV_1_ (%) and FEV_1_/FVC (%), compared with placebo. BY granule improved PEF. Both treatments reduced acute exacerbation frequency (P = 0.067), BODE index and psychological field score, while improved 6MWD. In terms of descent rang of SGRQ score, both treatments increased (P = 0.01). Both treatments decreased inflammatory cytokines, such as IL-8, and IL-17(P = 0.0219). BY granule obviously descended IL-17(P<0.05), IL-1β (P = 0.05), IL-6, compared with placebo. They improved the level of IL-10 and cortisol. BY granule raised cortisol (P = 0.07) and decreased TNF-α. Both treatments slightly descended TGF-β1. In terms of safety, subject compliance and drug combination, there were no differences (P>0.05) among three groups.

**Conclusions:**

BY granule and BF tablet were positively effective for the treatment of COPD, and the former performed better in general.

**Trial Registration:**

Chinese Clinical Trial Register center ChiCTR-TRC-09000530

## Introduction

Chronic obstructive pulmonary disease (COPD) is a complex inflammatory disease of airways with persistent airflow obstruction [1]. COPD is mainly caused by smoking, with lots of other pathogenic factors [2]. It is often diagnosed based on low airflow by lung function tests [3]. And this limitation is not fully reversible and commonly becomes worse over time. This disease is mainly observed in senile people. Management involves bronchodilators (β_2_ agonists and anticholinergics) [4], inhaled glucocorticosteroids [5], phosphodiesterase [6], leukotriene antagonists [7],statins [8], vaccinations, drug combination [9], quitting smoking [10], rehabilitation, long-term oxygen treatment or lung transplantation. Studies showed that present pharmacotherapy failed to reverse the downtrend in pulmonary function completely [11], [12]. At present COPD is still a major health problem, and its mortality is predicted to increase with a rise in smoking rates and an aging population in many countries [13].

However, COPD is considered to be treatable and preventable by Global Initiative for Chronic Obstructive Lung Disease (GOLD, 2013) [1]. Traditional herbal medicines may bring us new promises. People are increasingly paying close attention to it. A recent cross-sectional study indicated that one fifth patients with moderate to severe COPD had used some herbal preparation in Australia [14]. It is found that Traditional Chinese herbal medications (TCHM) can play an increasingly role for treating COPD according to literatures, which is a potential complementary and alternative therapy (CAT) for COPD [14]–[17]. But it may be important for us to find out its preponderant targets and links, thus improving the efficacy of traditional Chinese medicine (TCM) in prevention and treatment of COPD. As a complementary medicine, the effects of TCHM for treating COPD are not generally approved beyond any reasonable doubt. Currently, the evidence from randomized clinical trials is rarely known [18].

This study aims to evaluate the performance of TCHM treatment for COPD in Chinese population.

## Methods

The CONSORT checklist, trial protocol, and ethics approval document S1 are available as supporting information; see Checklist S1, Protocol S1, and Ethics Approval Document S1.

### Study participants

From December 30, 2010 to September 10, 2012, we enrolled patients with grade II or III (moderate to severe) stable COPD at five participating clinical centers according to Global Strategy for the Diagnosis, Management, and Prevention of COPD (2010) and Chinese Treatment Guidelines of Chronic Obstructive Pulmonary Disease (2009) (GOLD, 2010; COPD Study Group of Chinese Society of Respiratory Disease, 2009) on an initial medical evaluation. Additional inclusion criteria necessary for enrollment were an age limit ranged from 40 to 75 years, and the frequency of acute exacerbation each year ≥2.5 times in past two years. All patients conformed to the patterns of deficiency of qi or kidney qi or kidney yang according to syndrome differentiation of TCM (According to “Clinic terminology of traditional Chinese medical diagnosis and treatment-Syndromes, GB/T 16751.2-1997”). The research ethics boards of the participating hospitals (Huashan hospital of Fudan University, Xiyuan Hospital of CACMS, West China Hospital of Sichuan University, First Hospital Affiliated to Henan University of TCM, Jiangsu Province Hospital of TCM) approved the study, Ethics and the full study were approved by ethics committee of Huashan hospital, Fudan University in September 1 of 2009, and all patients signed written informed consents.

We excluded the patients with the following conditions: (1) Combination with bronchiectasis, pulmonary tuberculosis, diffusing panbronchiolitis, pulmonary interstitial fibrosis, pulmonary infection, pneumothorax, pleural effusion, pulmonary embolism, and neuromuscular disorders which affected the breathing movements. (2) Patients who were diagnosed with Deficiency of yin and abundance of fire in TCM. (3) Females who were pregnant or planed to be, and who were in lactation. (4) Patients with malignant tumor or blood diseases. (5) Those who had been involved in other clinical trials in the previous three months. (6) Those with severe damage to the heart, liver and kidney (heart function scale 3∼4, ALT and/or AST exceed the normal limit by more than 1.5 times, Cr surpasses the normal limit). (5) Those who were accompanied by increase of airway reactivity. (8) Other situations which the researchers believed to be inappropriate.

Before the treatment, the demographic information, physician examination information, accompanying symptoms, and medical history were recorded. We also measured biochemistry parameters, blood glucose profiles, blood lipids profiles, respiratory and heart rates, lung function parameters, and oxygen saturation percentage while the patients were breathing in room air.

The number of cases in the treatment group was the same with that of the control group. It was investigated that the effect of the treatment group was superior to that of the control group. The formulae were made according to superiority clinical trial sample size. In view of the previous therapeutic effects and statistical requirement, the follow-up loss rate should be controlled below 15–20%. The cases for observation from the qualified ones required by the treatment group and the control group were both 120. There were 360 cases in total (include the drop-out cases).

### Study intervention

The stratified randomization was used through a computer-generated random listing of three studied assignments blocked in groups according to research centers and lung functions (50%≤FEV1/pre-value %<80%, 30%≤FEV1/pre-value %<50%), so as to make sure the 360 cases could be distributed into the treatment group and control group at randomization. Each group included 120 cases. All the cases should be told and treated by avoiding the influence of environment and other causative factors. Double-grade blinding was adopted. The first grade was the treatment corresponding to the numbers (blind codes); the second grade was the code name corresponding to the three groups (randomized as A, B and C). The blind codes of the two grades were separately sealed up. Both were in duplicate and separately deposited in different clinical research institutes or sponsors. All study medications were prepared by one central pharmacy, and Bushen Yiqi (BY) granule, Bushen Fangchuan (BF) tablet and placebo were identical in both taste and appearance and were packaged identically with a double-dummy method. Neither research staff nor patients were aware of the treatment assignments before or after randomization. Patients were randomly assigned to one of the three studied groups. Patients in BY granule group received BY granule (a bag per time, 2 times daily) and placebo BF tablet (5 tablets per time, 3 times daily) for 180 days all together. And patients in BF tablet group received BF tablet (5 tablets per time, 3 times daily) and placebo BY granule (a bag per time, 2 times daily) for 180 days all together. Patients in the placebo group received placebos of BY granule and BF tablet with identical appearance and dosage for 180 days. The study was based on additional conventional therapy (inhaled albuterol or long-acting bronchodilator) according to GOLD (2010).

### Outcome measures and follow-up

We obtained data for the outcome measures from the patients by visits or standardized telephone interviews with trained techniques on days 30, 90, 180 after starting the treatment and days 90, 180 after finishing the treatment. The primary outcome included pulmonary function test, the frequency and duration of acute exacerbation, and scores of St. George Respiratory Questionnaire (SGRQ). Pulmonary function test included measurements of vital capacity (VC), forced expiratory volume in one second as a percentage of the predicted value (FEV_1_%), forced expiratory volume in one second/forced vital capacity (FEV1/FVC %) pre- and post-bronchodilator, and peak expiratory flow (PEF). An acute exacerbation of COPD was defined as an unscheduled visit to a physician's office or a return to the emergency department because of exacerbating dyspnea after randomization. We assessed patients at 30, 90, 180, 270 and 360 days after randomization to determine whether a relapse had occurred or not. For every suspected relapse we contacted both the patient and the physician to ensure that the visit had been prompted by dyspnea and had been urgent and unscheduled, and we obtained a copy of the written medical record of the encounter. An adjudication committee whose members were unaware of the patients' treatment assignments confirmed that all relapses met the study definition of relapse. We distributed the handbooks of SGRQ (Dairy of Patients with COPD) to the enrolled patients during each follow-up (baseline, 30 days, 90 days, 180 days, 270 days), and withdrew them during the next follow-up.

Secondary outcomes included BODE (body-mass index, obstruction of airways, dyspnea, exercise capacity) index, six-minute walking distance (6MWD), psychological field score, inflammatory cytokines measurement and indexes of hypothalamus-pituitary-adrenal (HPA) axis function. The BODE Index is a composite marker of disease taken into consideration the systemic nature of COPD. 6MWT evaluates the distance a patient can usually walk on a horizontal surface in six minutes and reflects an individual's ability to perform daily physical activities. Psychological field score is evaluated with the WHOQOL-BREF questionnaire (WHO, 2004), and can reflect patients' anxiety and depression due to the chronic diseases [19]. Inflammatory factors involved in IL6, IL8, IL10, IL-17, IL-1β, TGF-β1 and TNF-α were measured with peripheral serum. We tested the value of cortisol about HPA-axis function with peripheral plasma.

Levels of routine blood test, routine urine test, liver and kidney function test (triglycerides, total cholesterol, low-density lipoprotein cholesterol, high-density lipoprotein cholesterol), blood glucose, ECG were examined at baseline, days 90, and days 180. Blood pressure and pulse were measured by research assistants with an automated monitor on the same schedule as the visits to primary care providers.

### Statistical analysis

The Kolmogorov-Smirnov test was used to determine whether or not continuous variables followed a normal distribution. Variables that were not normally distributed were log-transformed to approximately normal distributions prior to analysis. The results are expressed as the mean ± standard deviation (SD), or the median, unless otherwise stated. Differences among the three groups in baseline characteristics and outcome measures were compared with the use of analysis of variance for continuous variables and the χ^2^ test for categorical variables. Following analysis of variance, Fisher's least significant difference test was used to explore further and compare the mean of one group with the mean of another. The analysis of covariance was used for controlling potential confounding variables.

The number of patients needed for each group was estimated based on a two-sided type I error rate of 0.05 or less and a power of 80% to detect differences in curative parameters, such as lung function indexes, frequency of acute exacerbation, score of SGRQ, and inflammatory cytokines level between those receiving BY granule and those receiving placebo. Similar statistical power was available to detect a difference between those receiving BF tablet and those receiving placebo. The results were analyzed using the Statistical Package for Social Sciences for Windows, version 16.0 (SPSS, Chicago, IL, USA). The tests were two-sided, and a p value of <0.05 was considered significant.

## Results

**Figure 1:**
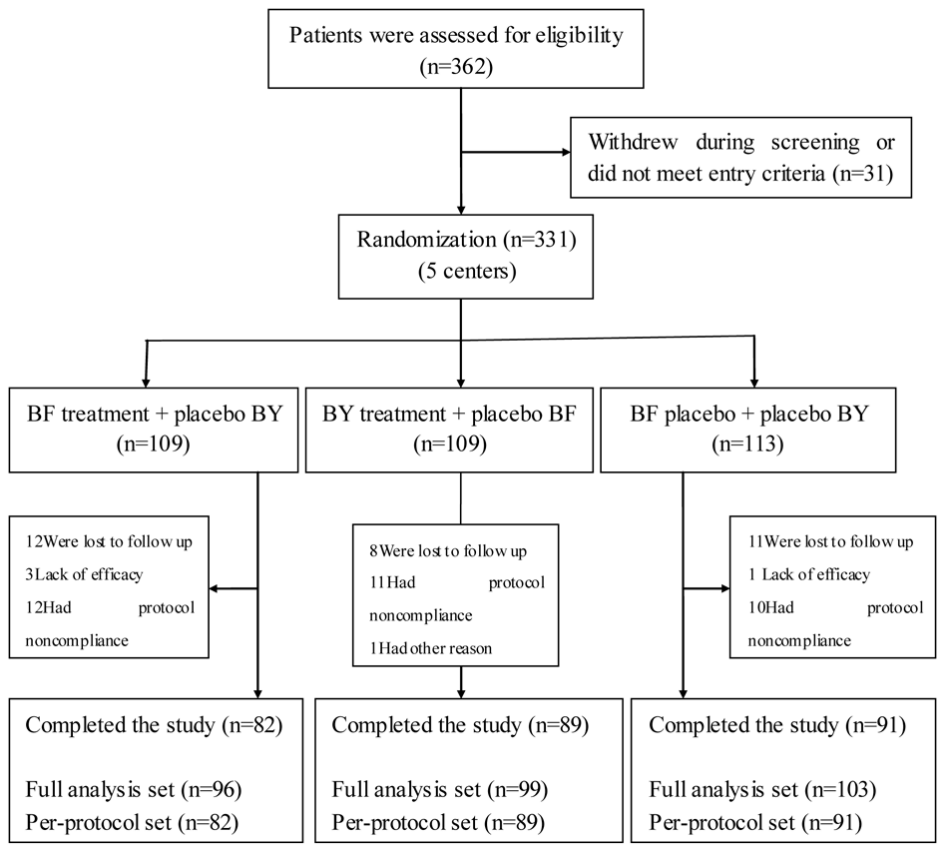
Enrollment of patients and completion of the study (Flow diagram).

A total of 362 potential patients were screened for the study, of whom 331 (91 percent) were eligible (Fig. 1). A total of 109 patients were assigned to receive BY granule, and 109 patients were assigned to receive BF tablet. A total of 113 patients were assigned to receive placebo. Of these 331 patients, 298 (90 percent) entered the full analysis set (FAS) at last. Among these excluded patients, 49 percent were ultimately hospitalized, 16 percent had received systemic corticosteroids in the emergency department or within the previous 30 days, and the rest were connected with no effective follow-up data for various reasons. A total of 262 patients were determined to meet the per-protocol set (PPS) finally.

The baseline clinical characteristics of patients were grouped according to treatment (Table 1). The entire sample included 195 men and 67 women (mean age 62.53±8.18 years; data not shown). Differences in weight were reported among the three groups. There was no significant difference in other baseline characteristics among the three groups.

**Table 1:** Baseline characteristics of the patients.

Characteristics	Per-protocol set			
	BF tablet group	BY granule group	Placebo group	P-value
	n = 82	n = 89	n = 91	
**Age**(year)	62.43±9.04	61.51±8.79	62.68±8.10	0.5039
**Height**(cm)	165.22±8.13	164.45±8.97	166.03±8.59	0.285
**Weight**(Kg)	61.81±9.41	60.89±11.45	64.49±11.81	0.0382
**Course of disease(month)**	12.57±8.39	12.75±8.95	12.43±9.46	0.9946
**Family history**				0.92
Yes	19(23.17%)	22(24.72%)	22(24.18%)	
No	63(76.83%)	67(75.28%)	69(75.82%)	
**Smoking history**				0.122
Yes	27(32.93%)	41(46.59%)	36(39.56%)	
No	55(67.07%)	47(53.41%)	55(60.44%)	
**Disease history**				0.145
Yes	25(30.49%)	18(22.22%)	32(35.96%)	
No	57(69.51%)	63(77.78%)	57(64.04%)	
**Medical history**				0.981
Yes	40(48.78%)	42(47.73%)	45(49.45%)	
No	42(51.22%)	46(52.27%)	46(50.55%)	
**Gende**r				0.333
Male	64(78.05%)	62(69.66%)	69(75.82%)	
Female	18(21.95%)	27(30.34%)	22(24.18%)	
**Blood pressure**				
Systolic pressure(mm/Hg)	121.84±10.06	122.09±8.10	120.88±9.62	0.6734
Diastolic pressure	77.36±5.58	76.40±6.00	76.40±7.27	0.3923
**Respiratory times(per minute)**	19.33±2.06	18.89±2.26	18.78±2.09	0.052
Temperature(°C)	36.64±0.27	36.67±0.23	36.67±0.25	0.7375
Heart rate**(per minute)**	79.43±10.78	76.82±6.82	77.79±8.66	0.1248
**Chest radiograph**				0.601
Normal	25(32.05%)	22(25.88%)	25(29.41%)	
Abnormal	53(67.95%)	63(74.12%)	60(70.59%)	
**Lung function**				
VC(L)	2.38±0.84	2.35±0.76	2.44±0.93	0.7528
FEV_1_(%)	46.65±15.55	48.47±15.44	46.30±15.14	0.5654
FEV_1_/FVC(%)pre	50.33±11.15	53.77±11.24	50.36±10.83	0.0898
FEV_1_/FVC(%)post	51.38±10.58	54.65±13.51	51.33±13.76	0.2968
PEF(L/S)	3.30±1.54	3.32±1.48	3.14±1.57	0.854
**GOLD Classification by Spirometry**				0.674
I(mild)	1(1.22%)	0(0.00%)	2(2.20%)	
II(moderate)	34(41.46%)	42(47.19%)	34(37.36%)	
III(severe)	45(54.88%)	44(49.44%)	50(54.95%)	
IV(very severe)	2(2.44%)	3(3.37%)	5(5.49%)	
**Differentiation of syndrome of TCM**				
qi deficiency				0.182
Yes	41(50.00%)	45(50.56%)	55(60.44%)	
No	41(50.00%)	44(49.44%)	36(39.56%)	
kidney qi deficiency				0.104
Yes	56(68.29%)	57(64.04%)	50(54.95%)	
No	26(31.71%)	32(35.96%)	41(45.05%)	
kidney yang deficiency				0.35
Yes	17(20.73%)	27(30.34%)	25(27.47%)	
No	65(79.27%)	62(69.66%)	66(72.53%)	
hyperactivity of fire due to yin				
Yes	0(0.00%)	0(0.00%)	0(0.00%)	
No	82(100.0%)	89(100.0%)	91(100.0%)	

**NOTE:** There are no significant differences (P>0.05) at the baseline of population (index of population: sex, age, height, weight, course of disease, family history, smoking history, past disease history, past medication history), general physical examination (BP, respiratory times, chest radiograph, temperature, heart rate), syndrome differentiation of TCM, and COPD classification, besides weight (P<0.05 BY group for placebo group).

### Primary outcomes

**Figure 2:**
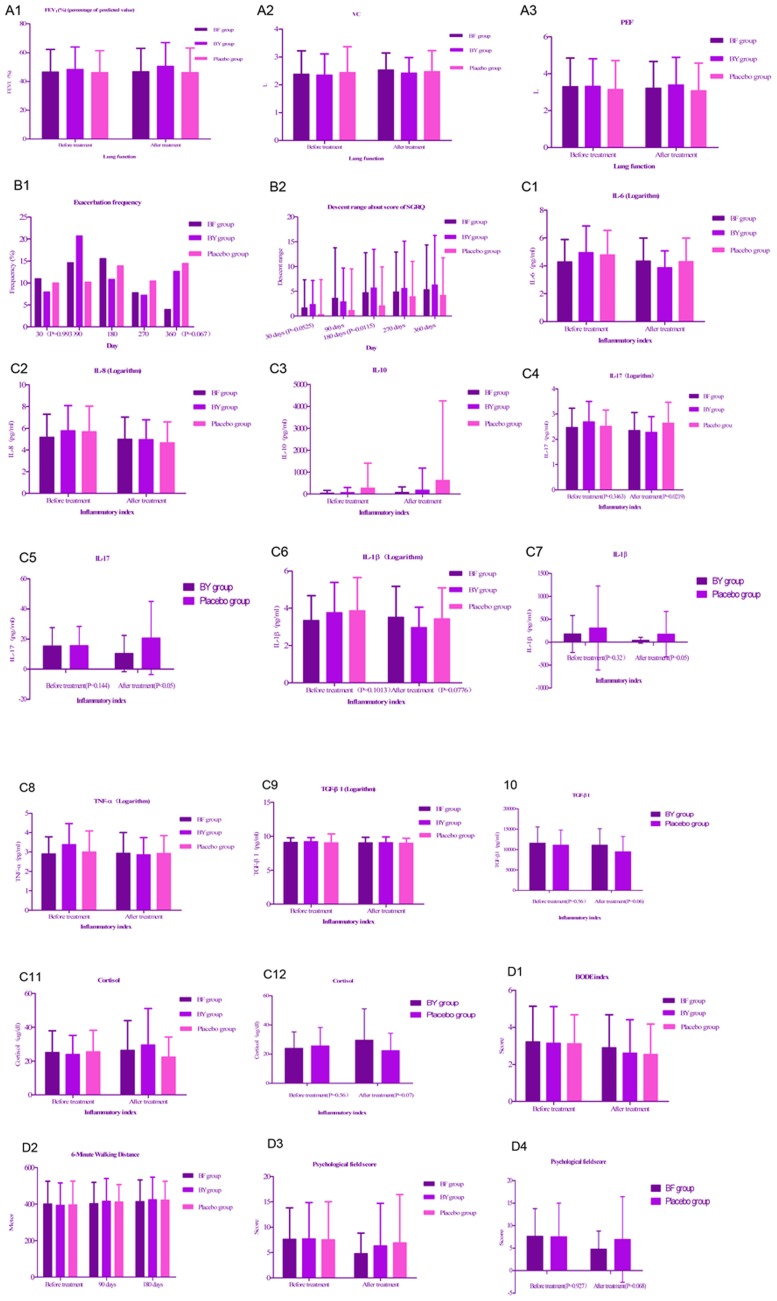
Statistical graphs. Note: Lung function indexes (FEV_1_ (%), VC, PEF) (A1∼3); Exacerbation frequency(%) (B1); Descent range about score of SGRQ (B2); Inflammatory indexes: IL6(C1), IL8(C2), IL10(C3), IL17(C4∼5), IL-1β(C6∼7), TNF-α(C8), TGF-β1(C9), Cortisol(C11∼12); BODE index(D1); 6MWD (D2); Psychological field score (D3∼4).

Compared with placebo, BF tablet increased the values of VC, FEV_1_ (%), and FEV_1_/FVC (%) in pulmonary function test. Similar results were reported in BY granule group and it additionally had a very good uptrend about the value of PEF. (Figure 2: A1∼3; Table 2). The proportion of patients with acute exacerbation of COPD was on the rise ranging from 10.00% to 14.46% in placebo group, whereas that of both active treatment groups began to continue a downtrend after 180 days. The proportion with acute exacerbation decreased from 10.98% of 1^st^ follow-up to less than 5% of 6^th^ follow-up in BF tablet group. The P-value of the exacerbation frequency (%) was 0.067 among three groups at 360 days. (Figure 2: B1; Table 2). In terms of the descent range about SGRQ score, there were always increasing values in the active treatments in contrast with placebo. Particularly, there was a significant difference among the three groups after 30 days of treatment (P = 0.05), and a very significant difference after 180 days of treatment (P = 0.01) (Figure 2: B2).

**Table 2:** Variable of lung function, Frequency of acute exacerbation, and Score and descent range of SGRQ before and after treatment.

Variables	Per-protocol set			
	BF tablet group	BY granule group	Placebo group	P-value
	n = 82	n = 89	n = 91	
**Lung function**				
VC(L)				
Before treatment	2.38±0.84	2.35±0.76	2.44±0.93	0.7528
After treatment	2.53±0.61	2.42±0.56	2.47±0.76	0.7077
FEV1(%) (percentage of predicted value)				
Before treatment	46.65±15.55	48.47±15.44	46.30±15.14	0.5654
After treatment	46.91±16.10	50.57±16.39	46.30±16.92	0.2307
FEV1/FVC (%) (pre-bronchodilator)				
Before treatment	50.33±11.15	53.77±11.24	50.36±10.83	0.0898
After treatment	52.47±11.97	55.39±13.01	53.32±15.06	0.6103
FEV1/FVC (%) (post-bronchodilator)				
Before treatment	51.38±10.58	54.65±13.51	51.33±13.76	0.2968
After treatment	54.50±13.14	57.47±14.96	54.85±15.28	0.4328
PEF(L/S)				
Before treatment	3.30±1.54	3.32±1.48	3.14±1.57	0.854
After treatment	3.21±1.45	3.39±1.49	3.08±1.50	0.5612
**Frequency of acute exacerbation**				
at 30days				0.993
0	73(89.02%)	81(92.05%)	81(90.00%)	
once	9(10.98%)	5(5.68%)	9(10.00%)	
twice	0(0.00%)	1(1.14%)	0(0.00%)	
≥three times	0(0.00%)	1(1.14%)	0(0.00%)	
at 90days				0.62
0	70(85.37%)	65(79.27%)	79(89.77%)	
once	12(14.63%)	17(20.73%)	7(7.95%)	
twice	0(0.00%)	0(0.00%)	2(2.27%)	
≥three times	0(0.00%)	0(0.00%)	0(0.00%)	
at 180days				0.785
0	65(84.42%)	74(89.16%)	74(86.05%)	
once	12(15.58%)	8(9.64%)	12(13.95%)	
twice	0(0.00%)	1(1.20%)	0(0.00%)	
≥three times	0(0.00%)	0(0.00%)	0(0.00%)	
at 270days				0.851
0	71(92.21%)	77(92.77%)	77(89.53%)	
once	6(7.79%)	5(6.02%)	9(10.47%)	
twice	0(0.00%)	1(1.20%)	0(0.00%)	
≥three times	0(0.00%)	0(0.00%)	0(0.00%)	
at 360days				0.067
0	72(96.00%)	69(87.34%)	71(85.54%)	
once	3(4.00%)	10(12.66%)	10(12.05%)	
twice	0(0.00%)	0(0.00%)	2(2.41%)	
≥three times	0(0.00%)	0(0.00%)	0(0.00%)	
**Score of SGRQ and descent range**				
Score before treatment	37.23±15.23	38.11±15.74	36.67±15.51	0.7602
Descent range of 30days	1.64±5.69	2.35±4.82	0.30±7.05	0.0525
Score at 30days	35.51±14.88	36.18±14.95	36.23±14.53	0.7778
Descent range of 90days	3.62±10.13	2.88±6.81	1.15±8.38	0.1321
Score at 90days	34.18±13.88	35.02±14.64	36.57±15.75	0.2358
Descent range of 180days	4.73±8.02	5.65±7.82	2.07±7.85	0.0115
	P<0.05 for BF VS Placebo, P<0.05 for BY VS Placebo, P>0.05 for BF VS BY			
Score at 180days	33.66±15.74	33.13±14.53	34.56±15.13	0.6384
Descent range of 270days	4.87±8.05	5.59±9.52	3.92±7.09	0.508
Score at 270days	33.34±14.88	33.09±13.72	32.53±15.09	0.9976
Descent range of 360days	5.30±9.05	6.29±9.98	4.21±7.53	0.36
Score at 360days	33.07±14.86	32.91±14.05	33.10±14.49	0.9487

### Secondary outcomes

The values of inflammatory factors of IL-8 and IL-17 decreased in both of the active treatment groups. Different from BF tablet group, BY granule group had the declining trend of IL-1β and TNF-α. Importantly, BY granule group obviously descended the level of IL-17 (P<0.05), IL-1β (P = 0.05), and IL-6, in contrast with placebo group (Figure 2: C1–2, C5∼8; Table 3). Both the active treatments slightly descended TGF-β1 compared with placebo, whereas the P-value about TGF-β1 was 0.06 after treatment between BY granule group and placebo group (Figure 2: C9–10; Table 3). There were increasing values of inhibition-inflammatory factor of IL-10 in both active-treatment groups (Figure 2: C3; Table 3). Similarly, the values of cortisol were found to increase in both active-treatment groups, and there was one P-value 0.07 of cortisol between BY granule and placebo (Figure 2: C11–12; Table 3). The scores of BODE index and psychological field were found to reduce in both active-treatment groups, while the indicators of 6MWD were reported to increase (Figure 2: D1–3); Table 3. Especially BF tablet group had the p-value 0.068 of psychological field score as compared with placebo (Figure 2: D4; Table 3).

**Table 3:** Expressions of blood cytokines, Score of BODE, 6MWD, and Psychological field before and after treatment.

Variables	Per-protocol set			
	BF tablet group	BY granule group	Placebo group	P-value
	n = 82	n = 89	n = 91	
IL-6 (logarithm)				
Before treatment	4.29±1.60	4.96±1.90	4.79±1.77	0.0912
After treatment	4.34±1.66	3.87±1.21	4.31±1.68	0.1776
IL-8 (logarithm)				
Before treatment	5.18±2.11	5.79±2.30	5.69±2.35	0.1098
After treatment	5.00±2.03	4.96±1.83	4.67±1.92	0.7213
IL-10				
Before treatment	48.00±117.16	73.73±223.03	269.81±1135.22	0.1668
After treatment	81.01±241.75	180.41±995.53	625.43±3620.71	0.4112
IL-17 (logarithm)				
Before treatment	2.47±0.76	2.69±0.81	2.52±0.64	0.3463
After treatment	2.35±0.71	2.28±0.62	2.64±0.83	0.0219
	P<0.05 for A VS C, P<0.05 for BY VS Placebo			
IL-1β(logarithm)				
Before treatment	3.35±1.33	3.77±1.61	3.87±1.78	0.1013
After treatment	3.52±1.65	2.96±1.09	3.43±1.67	0.0776
TGF-β1 (logarithm)				
Before treatment	9.13±0.66	9.23±0.59	9.06±1.28	0.6813
After treatment	9.04±0.81	9.07±0.84	9.01±0.71	0.9174
TNF-α(logarithm)				
Before treatment	2.91±0.87	3.39±1.07	3.00±1.08	0.0563
After treatment	2.94±1.06	2.86±0.88	2.93±0.92	0.8333
Cortisol				
Before treatment	25.23±12.73	23.97±11.27	25.61±12.67	0.5928
After treatment	26.46±17.49	29.59±21.48	22.43±11.85	0.2123
**BODE score**				
Before treatment	3.22±1.91	3.14±1.98	3.12±1.56	0.8635
After treatment	2.90±1.78	2.61±1.80	2.54±1.63	0.3907
**6MWD score**				
Before treatment	401.16±123.85	394.48±121.99	396.27±130.34	0.8416
90days	403.88±115.52	416.01±124.73	412.34±94.58	0.6493
180days	414.95±117.45	424.12±123.83	422.74±103.11	0.8462
**Psychological field score**				
Before treatment	7.63±6.16	7.71±7.13	7.54±7.47	0.994
After treatment	4.78±4.02	6.30±8.40	6.92±9.50	0.1012

In terms of subject compliance and drug combination, there were no statistic differences among the three groups (P>0.05). The occurred population ratios with good compliance were 81.25%, 88.89%, and 82.52% in BF tablet group, BY granule group and placebo group, respectively. And those with drug combination were 44.79%, 41.41%, and 50.49% in BF tablet group, BY granule group and placebo group, respectively.

### Adverse events

The percentage of patients with adverse events was 13.54%, 14.14% and 16.50% in BF tablets group, BY granules group and placebo group, respectively. There was no differences among the three groups (P>0.05). No serious adverse event was reported in this study.

## Discussion

In modern medicine, spirometry is required to establish a diagnosis of COPD [1]. The tendency of increasing VC, FEV_1_ (%), PEF and FEV_1_/FVC (%) in the two treatment groups compared with placebo after six-month therapy, provided evidence that they might postpone the downtrend of pulmonary function and improve the life quality of COPD patients. Whereas those results about exacerbation frequency, BODE index, psychological field scores, six-minute walking distance and descent range of SGRQ scores, indicated that the two active treatments could improve daily physical activities and decrease the risk of adverse health events such as acute exacerbations.

Inflammation always follows after the disease of COPD, so the anti-inflammation therapy is one of the critical treatments for COPD. We found that BY granule and (or) BF tablet could decrease these pro-inflammatory cytokines, such as IL-6, IL-8, IL-1β, TNF-α and IL-17. They characterized of improving the level of IL-10 and cortisol. These findings supported the hypothesis that the two treatments, especially BY granule, benefited to patients with COPD in clinical practice. Interestingly, TGF-β1 could delay the development of COPD, and help repair damage to the lungs of individuals who are at risk of developing COPD, preserving the decline of FEV_1_ [20], [21]. Recent researches provided the levels of TGF-β1 were different at four stages of COPD with highest in stage IV, and inversely correlated with FEV_1_ (% predicted) and FVC (% predicted) [22], [23]. Our study showed a slight decline in both active treatment groups after 180 days. It might be a scientific and reasonable result, and deserved further detection.

The data of safety, subject compliance and drug combination, told us that the two Chinese herbal formulae are relatively safe with fewer side-effects, which is different from western medicines [15].

Our findings were consistent with other clinical reports about TCHMs worldwide [16], [18], [24]. TCHMs were effective for the treatment of COPD, improving lung function and quality of life, reducing the frequency and duration of acute exacerbation, ameliorating symptoms, and so on. But there were few multicenter, double-blind, randomized controlled trials of stable COPD with herbal medicines. The sample of our study was not only relatively large in size, but also covered different regions of north and south China. Different from others, moreover, our study had three groups with double-dummy medications, and included observation of HPA-axis function and inflammation indexes. The results of blood samples should be more objective and persuasive than these of others.

For treating COPD, although at present it was possibly more effective to use an inhaled corticosteroid combined with a long-acting beta_2_-agonist than using a single component, combination therapy was associated with an increased risk of pneumonia [25]–[27]. However, no relevant risk was found in our study. This might be one of particular benefits in Chinese herbal medicines compared with western medicines.

**Figure 3:**
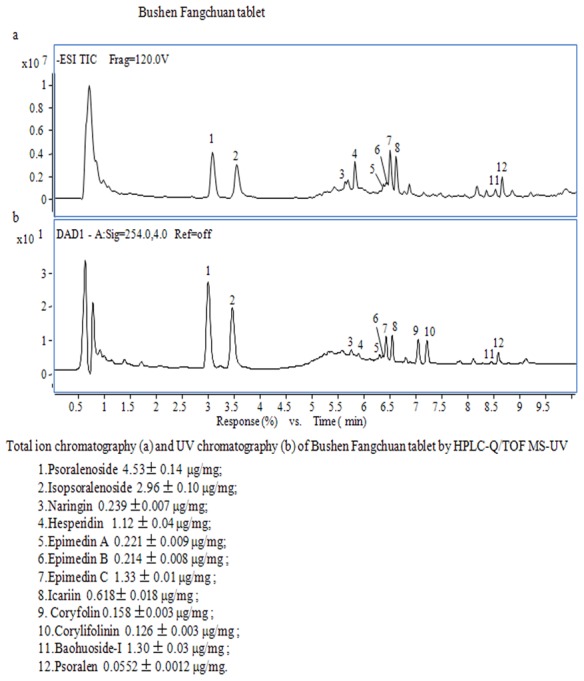
Total Ion chromatography, UV chromatography and content of main compositions of the preparation Bushen Fangchuan tablet by HPLC-Q/TOF MS-UV.

BF tablet is a patent medicine and TCM preparation, made from eight kinds of Chinese herbal medicines. It can prevent cough, eliminate phlegm, and relieve asthma according to animal tests. It also has strong anti-inflammation effect. Clinically, the TCM effects of BF tablet are mainly warming yang and tonifying the kidney, used for preventing seasonal onset of asthma, chronic bronchitis, and cough (Table 4, Figure 3).

**Table 4:** Introduction to treated drugs of TCH.

Drug name	Name of Chinese Herbal Medications of the drug	Compound	Molecular Formula of the Compound	Molecular Weight	Main Efficacy of TCM
Bushen Yiqi granule, TCM agreement prescription, 5 g/bag equal to 32.5 g of decoction pieces. Batch numbers 1006380, 1006381, 1006382	Radix Astragaliseu Hedysari	Astragaloside	C_41_H_68_O_14_	784.9702	Tonifying kidney and qi, relieving asthma
	Herba Epimedii	Icariin	C_33_H_40_O_15_	676.65	
		Epimendin B	C38H48O19	808.78	
		Epimendin C	C39H50O19	822.80	
	Radix Rehmanniae Recens	Catalpol	C_15_H_22_O_10_	362.33	
Bushen Fangchuan Tablet, China Patent medicine, 0.25 g/tablet, Batch number 10100006	Made from eight kinds of Chinese herbal medications including Herba Epimedii, Radix Rehmanniae Recens, Prepared Lateral Root of Aconite, psoralea corylifolia, the seed of Chinese dodder, prepared radix rehmanniae, Chinese yam, Orange peel.				Warming yang, Tonifying kidney
	Approved by China: Z50020405				

**Figure 4:**
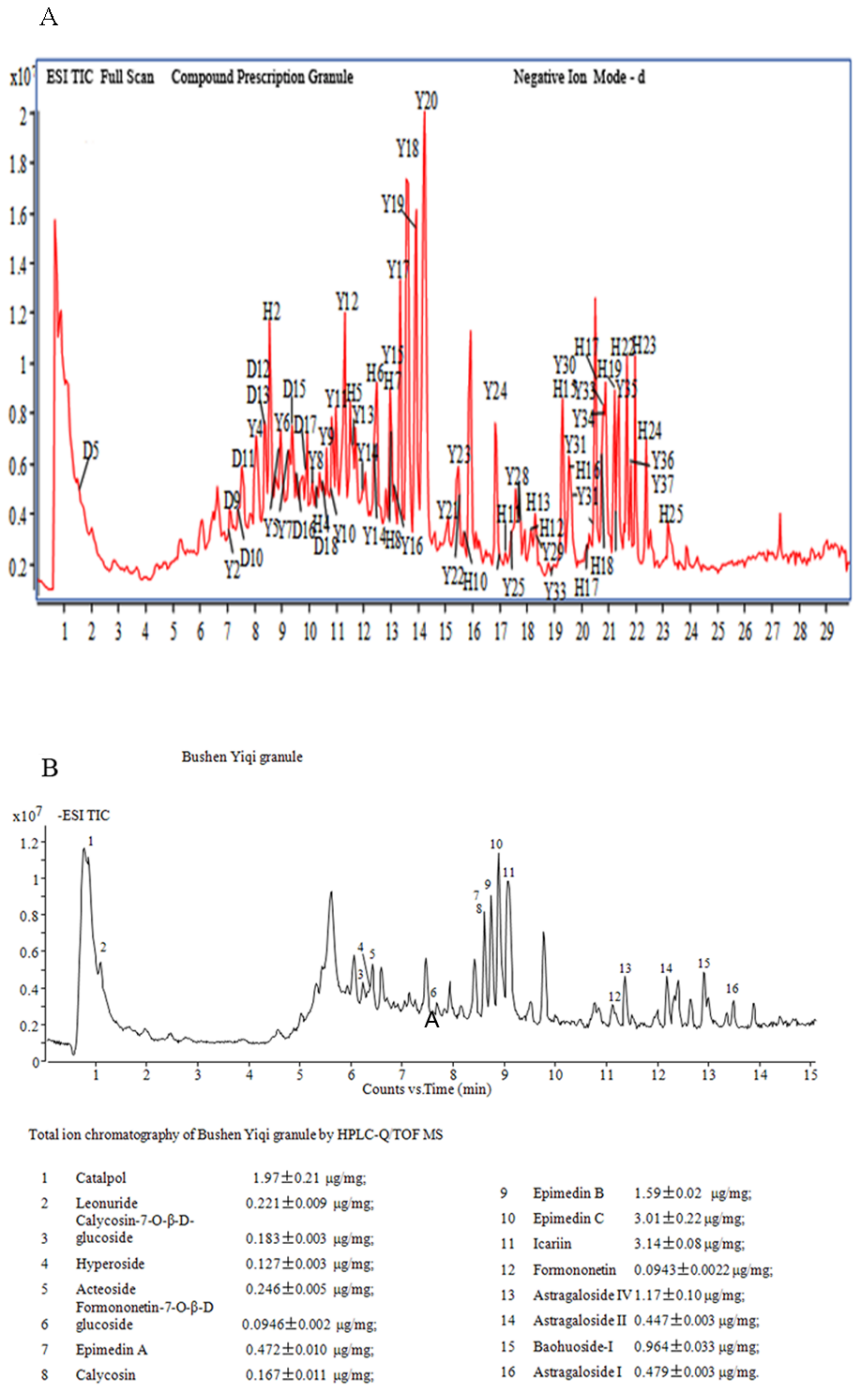
Mass Spectroscopy of Bushen Yiqi granule. A. Analysis and appraisal of chemical compositions for the preparation Bushen Yiqi granule with the method of LC-ESI-Q-TOF-MS/MS. **Note:** The results showed 71 chemical compositions of Bushen Yiqi granule: Herba Epimedii(35), Astragaloside(23), Radix Rehmanniae Recens(13). B. Ion chromatogram and content of main compositions of the preparation Bushen Yiqi granule. **Note:** The results showed Icariin had the highest content, and then Epimendin C, Catalpol, Epimendin B, Astragaloside in order among chemical compositions of Bushen Yiqi granule.

BY granule is an agreement prescription and compound preparation, made from three kinds of Chinese herbals. Clinically, the TCM effects of BY granule are mainly tonifying the kidney, replenishing qi and relieving asthma. The results of mass spectroscopy showed 71 bioactive compositions in the formula like Icariin, Astragaloside, Catalpol, and so on (Table 4–5, Figure 4). Its effect was certainly connected with these chemical compositions, which had such activities as regulating HPA axis function, anti-inflammation, adjusting immune disfunction, relieving edema, and antioxidation [28]–[31]. In this study, we thought that different dosage forms might be a reason for the inferior effect of BF tablet compared with BY granule, or the latter performed practically better for the treatment of COPD than the former. There was no Radix Astragaliseu Hedysari in BF tablet, which might be also a critical factor for its inferior efficacy compared with BY granule.

**Table 5:** Content of main compositions of the preparation Bushen Yiqi granule.

NO	Compound	Batch1	Batch2	Mean
		(ug/mg granule)	(ug/mg granule)	(ug/mg granule)
1	Formononetin	0.0943±0.0022	0.0979±0.0038	0.0961
2	Calycosin	0.167±0.011	0.172±0.004	0.17
3	Motherwort glycosides	0.221±0.009	0.187±0.006	0.204
4	Catalpol	1.97±0.21	2.05±0.02	2.01
5	Hyperoside	0.127±0.003	0.134±0.003	0.131
6	Ononin	0.0946±0.002	0.0939±0.001	0.0942
7	Calycosin glycosides	0.183±0.003	0.166±0.001	0.175
8	Bao beans glycosides	0.964±0.033	1.21±0.04	1.08
9	Verbascoside	0.246±0.005	0.319±0.004	0.283
10	Icariin	3.14±0.08	2.60±0.04	2.87
11	Astragaloside	1.17±0.10	1.12±0.00	1.15
12	Epimendin B	1.59±0.02	1.49±0.02	1.54
13	Epimendin C	3.01±0.22	2.65±0.02	2.83
14	Astragalosides	0.447±0.003	0.482±0.003	0.465
15	Epimendin	0.472±0.010	0.504±0.007	0.488
16	Astragalosides	0.479±0.003	0.488±0.003	0.484

**Note:** The results showed Icariin had the highest content, and then Epimendin C, Catalpol, Epimendin B, Astragaloside in order among chemical compositions of Bushen Yiqi granule.

**Figure 5:**
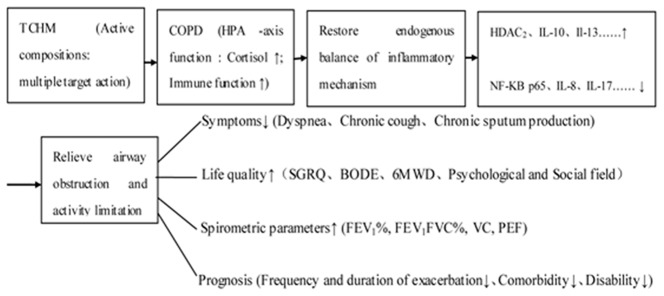
A possible biological mechanism of TCHMs for intervening COPD.

In our COPD model studies, we also found BY granule was provided with the results of anti-inflammation and antioxidation, also further proving BY granules' efficacy for treating COPD. We thought it was possibly one important pathogenesis for COPD breaking of the endogenous balance about generation and inhibition of inflammation factors. So a biological mechanism of TCHMs for intervening COPD might be as following simple sign with reference to the results of our model studies before (Figure 5).

The limitations of this study deserve comment. The 12-month follow-up and 6-month treatment period was not long enough, so quite a few promising results came out with no statistic difference in our study. Moreover, there was no stratified–method comparison based on the basic treatment because of some additional conventional drugs unrecorded due to negligence during the test at some research centers. More standard and scientific measures should be carried out for further research in future.

## Conclusions

This study showed that these two traditional Chinese herbal formulae BY granule and BF tablet were positively effective for the treatment and prevention of COPD. In general, BY granule performed better than BF tablet for treating COPD. Demonstrably, TCHM is promising, as complementary and alternative medicine (CAM), which may make up for the shortness of conventional drugs about COPD at present. Therefore, further studies related to TCHM are necessary and urgent for treating COPD in future.

### Patient consent

Obtained.

### Ethics approval

Ethics committees of Huashan hospital of Fudan University, Xiyuan Hospital of CACMS, West China Hospital of Sichuan University, First Hospital Affiliated to Henan University of TCM, Jiangsu Province Hospital of TCM.

A completed **CONSORT checklist** was provided.

## Supporting Information

Checklist S1 **CONSORT Checklist.**
(DOC)

Protocol S1 **Trial Protocol.**
(DOC)

Ethics Approval Document S1 **Ethics approval document.**
(DOC)
