# Correction: Effects of Two Chinese Herbal Formulae for the Treatment of Moderate to Severe Stable Chronic Obstructive Pulmonary Disease: A Multicenter, Double-Blind, Randomized Controlled Trial

**DOI:** 10.1371/journal.pone.0152379

**Published:** 2016-03-22

**Authors:** Genfa Wang, Baojun Liu, Yuxue Cao, Yijie Du, Hongying Zhang, Qingli Luo, Bei Li, Jinfeng Wu, Yubao Lv, Jing Sun, Hualiang Jin, Kai Wei, Zhengxiao Zhao, Lingwen Kong, Xianmei Zhou, Qing Miao, Gang Wang, Qingwei Zhou, Jingcheng Dong

A reference is omitted from the seventh sentence of the first paragraph under the subheading “Outcome measures and follow-up” in the Methods.

The sentence should read: An adjudication committee whose members were unaware of the patients’ treatment assignments confirmed that all relapses met the study definition of relapse (Aaron et al., 2003).

The reference is: Aaron SD, Vandemheen KL, Hebert P, Dales R, Stiell IG, Ahuja J, et al. Outpatient Oral Prednisone after Emergency Treatment of Chronic Obstructive Pulmonary Disease. N Engl J Med 2003; 348:2618–25.
