# Supplementary material for: Effects of Two Chinese Herbal Formulae for the Treatment of Moderate to Severe Stable Chronic Obstructive Pulmonary Disease: A Multicenter, Double-Blind, Randomized Controlled Trial
Source: PLoS One. 2014 Aug 13;9(8):e103168. doi: 10.1371/journal.pone.0103168 (PMC4132093; doi:10.1371/journal.pone.0103168)
Supplement: Protocol S1 — Trial Protocol. (DOC) [file pone.0103168.s002.doc]

**
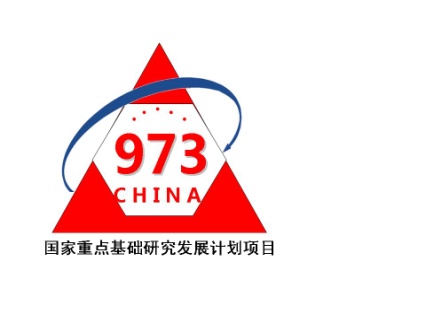
Trial protocol**

**The National Basic Research Program of China**

**（2009CB523001）**

**Clinical Study of Bushen Yiqi Prescription on Intervention of Various Inflammatory Diseases**

**Clinical Trial Protocol**

Effects of Bushen Yiqi Prescription and Bushen Fangchuan Tablet on Treating COPD: A Multicenter, Double-blind, Randomized Parallel Controlled Trial

| Application unit | Huashan Hospital Affiliated to Fudan University |
| --- | --- |
| Units in charge : | Huashan Hospital Affiliated to Fudan University |
| Participating units: | Xiyuan Hospital Affiliated to  China Academy of Chinese  Medical Sciences |
|  | Huaxi Hospital Affiliated to Sichuan University |
|  | The First Affiliated Hospital of Henan College of Traditional Chinese Medicine |
|  | Jiangsu Provincial Hospital of Traditional Chinese Medicine |
| Unit for trial design | Huashan Hospital Affiliated to Fudan University |
| Trial designers | Dong Jingcheng, Cao Yuxue |
| Date of enactment | 2009.9.3 |

**Name and phone number of the researchers**

|  | **Affiliation** | **Name** | **Phone** |
| --- | --- | --- | --- |
| Principal of the project | Huashan Hospital Affiliated to Fudan University | Dong Jingcheng | 13601761761 |
| Principal of the branch centers | Xiyuan Hospital Affiliated to China Academy of  Chinese Medical Sciences | Mao Qing | 13910812309 |
| Principal of the branch center | Huaxi Hospital Affiliated to Sichuan University | Wang Gang | 13228210301 |
| Principal of the branch center | The First Affiliated Hospital of Henan College of Traditional Chinese Medicine | Zhou Qingwei | 13700846704 |
| Principal of the branch center | Jiangsu Provincial Hospital of Traditional Chinese Medicine | Zhou Xianmei | 13951035477 |
| Researcher of the project | Huashan Hospital Affiliated to Fudan University | Duan Xiaohong | 13023208106 |
| Researcher of the project | Huashan Hospital Affiliated to Fudan University | Cao Yuxue | 13564199471 |
| Researcher of the project | Huashan Hospital Affiliated to Fudan University | Zeng Lixuan | 13124816951 |
| Researcher of the project | Huashan Hospital Affiliated to Fudan University | Li Lulu | 13816862497 |
| Researcher | Huashan Hospital Affiliated to Fudan University | Wu Jinfeng | 021-52888301 |
| Researcher of the project | Huashan Hospital Affiliated to Fudan University | Zhang Siwei | 021-52889999 |
| Researcher of the project | Huashan Hospital Affiliated to Fudan University | Chen Xiaodong | 021-52889999 |
| Researcher of the project | Xiyuan Hospital Affiliated to China Academy of Chinese Medical Sciences | Zhang Wenjiang | 13136139157 |
| Researcher of the project | Huaxi Hospital Affiliated to Sichuan University | Wang Lei | 13208160858 |
| Researcher of the project | The First Affiliated Hospital of Henan College of Traditional Chinese Medicine | Song Dongsheng | 13523512578 |
| Researcher of the project | The First Affiliated Hospital of Henan College of Traditional Chinese Medicine | Li Bin | 13938444286 |
| Researcher of the project | Jiangsu Provincial Hospital of Traditional Chinese Medicine | Zhu Jiping | 13222009558 |
| Researcher of the project | Jiangsu Provincial Hospital of Traditional Chinese Medicine | Xia Jun | 13851475757 |

**Flow Sheet of the Trial**

| Stage | | Start up | Medication | | | | Discontinuation and follow up | |
| --- | --- | --- | --- | --- | --- | --- | --- | --- |
| Interview | | 0 | 1 | 2 | 3 | 4 | 5 | 6 |
| Time | | -2W | M1±3d | M2±3d | M4±3d | M7±3d | M10±3d | M13±3d |
| **Collection of basic disease history** | | | | | | | | |
| Sign the informed consent | |  | **√** |  |  |  |  |  |
| Confirm the inclusion | |  | **√** |  |  |  |  |  |
| Write the general data | | **√** |  |  |  |  |  |  |
| History of past illness and history of treatment | | **√** |  |  |  |  |  |  |
| Drug combination and basic medication | | **√** | **√** | **√** | **√** | **√** | **√** | **√** |
| Screening indexes | X-ray film |  | **√** |  |  |  |  |  |
|  | Airway reactivity test | **√** |  |  |  |  |  |  |
| **Observation of effects** | | | | | | | | |
| Frequency and internal of acute exacerbation | |  | **√** | **√** | **√** | **√** | **√** | **√** |
| St George’s questionnaire | |  | **√** | **√** | **√** | **√** |  |  |
| TCM syndrome score | |  | **√** | **√** | **√** | **√** |  |  |
| Inflammation index | |  | **√** |  |  | **√** |  |  |
| HPA axis function | |  | **√** |  |  | **√** |  |  |
| P | |  | **√** |  |  | **√** |  |  |
| BODE index | |  | **√** |  |  | **√** |  |  |
| 6MWT | |  | **√** |  |  | **√** |  |  |
| Psychological stress scale | |  | **√** |  |  | **√** |  |  |
| **Observation on safety** | | | | | | | | |
| Routine blood and urine tests | |  | **√** |  | **√** | **√** |  |  |
| Functions of the liver and kidney, blood sugar | |  | **√** |  | **√** | **√** |  |  |
| Electrocardiogram | |  | **√** |  | **√** | **√** |  |  |
| **Other work** | | | | | | | | |
| Record adverse events | |  |  | **√** | **√** | **√** |  |  |
| Calculate the cases of drop-out | |  | **√** | **√** | **√** | **√** |  |  |
| Calculate the quantity of drugs | |  | **√** | **√** | **√** | **√** |  |  |
| Distribute the drugs | |  | **√** | **√** | **√** |  |  |  |
| Paste the examination report | |  | **√** |  | **√** | **√** |  |  |

**Catalogue**

1. Background and Objective

2. Case selection

2.1 Research subject

2.2 Diagnostic criteria

2.2.1 Diagnostic criteria of COPD

2.2.2 COPD staging

2.2.3 COPD Criteria for grading of severity

2.2.4 Evaluation of dyspnea

2.2.5 Diagnostic criteria for airway hyperreactivity

2.2.6 TCM criteria for syndrome differentiation

2.3 Inclusion criteria

2.4 Exclusion criteria

2.5 Deletion criteria

2.6 Criteria and treatment for drop-outs

2.7 Trial termination criteria

3. Research method

3.1 Research design

3.2 Sample size

3.3 Randomized grouping

3.4 Blind method

3.4.1 Blind design

3.4.2 Requirements for unblinding and emergent blindness-breaking

4. Treatment therapy

4.1 Preparation of trial medicines

4.1.1 Name, specification, usage and dosage of the medicines

4.1.2 Package of the medicines

4.1.3 Preparation of the drug label

4.2 Allocation of blind codes for the drugs

4.3 Allocation of drugs

4.4 Compliance

4.5 Combination of drugs

4.6 Storage of drugs

4.7 Start-up stage

4.8 Disease course and interview

5. Observation indexes and methods

5.1 Indexes for assessment of clinical therapeutic effects

5.1.1 Primary indexes for therapeutic effects

5.1.2 Secondary indexes for therapeutic effects

5.2 Evaluation of safety

5.2.1 General evaluation

5.2.2 Adverse reactions

6. Clinical observation and follow-up steps

6.1 The first visit (-W2)

6.2 The first interview (baseline) (M1)

6.3 The second interview (M2)

6.4 The third interview (M4)

6.5 The forth interview (M7)

6.6 The fifth, sixth interviews (M10, M13)

7. Criteria for evaluation of therapeutic effect

7.1 Criteria for evaluation of therapeutic effect with modern medical indexes

7.2 TCM criteria for evaluation of therapeutic effect

8 Contents of statistical analysis

8.1 Concepts

8.2 Analysis of completion situation

8.3 Analysis index of baseline balance

8.4 Indexes for analysis of therapeutic effect

8.6 Statistical analysis method

9 Management of the data

9.1 The writing and transfer of case report

9.2 The design and establishment of database

9.3 Data coding

9.4 Data entry

9.5 The audit of data

9.6 data locking

9.7 Data storage

10 Quality control and guarantee of the clinical trial

10.1 Measures for quality control

10.1.1 Quality control in the laboratory

10.1.2 Qualification inspection of researchers

10.1.3 Training before the clinical trial

10.1.4 Compliance

10.1.5 Adverse reactions

10.2 Measures of quality control

11. Predicted progress and completion time

11.1 Start-up time of the clinical trial

11.2 Mid-term coordination meeting

11.3 The completion time of the trial

11.4 The time of collecting, analyzing and summarizing of the trial data

Attachment

1. Emergency treatment plan of acute exacerbation of chronic obstructive pulmonary disease (AECOPD)

2. ST. George’s respiration questionnaire; please refer to COPD journals

3 BODE indexes and evaluation

4. Quantitative scores of TCM syndrome

5. Standard operating procedures of 6MWT

6. The process and collection of blood samples

7. Education for patients with COPD

8．References

**1. Background and Objective**

Chronic objective pulmonary disease (COPD) is characterized by unsmooth airflow, which is not fully reversible, progressive, and related to abnormal inflammation reaction of the lung to hazardous gases or particles produced by smoking or other factors.

COPD is chronic and complicated. Chronic bronchitis is believed to be the main cause for it. This disease is mainly seen in senile people. TCM holds that it is mainly due to deficiency of the lung and kidney, and Chinese medicine can be effective for this disease. It is necessary to take advantage of the holistic nature of TCM to prevent and treat COPD. Bushen Fangchuan Pian (Kidney-nourishing and Asthma-preventing Tablet) is an effective prescription for COPD with kidney deficiency, which is developed through years of our clinical investigation and research. This prescription is composed of Huangqi, Xianlingpi and other Chinese medicines. This proved prescription is effective in nourishing the kidney, replenishing qi and relieving asthma. Earlier research showed that this prescription can improve the HPA axis function, regulate the body immunization, and regulate the lung function of the rats with COPD and asthma. Clinically it can reduce the chances of acute exacerbation, enhance the motive ability, decrease the levels of peripheral blood inflammatory cytokines, improve life quality and promote the immune function of the patients.

In this research, we observed the patients’ frequency and degree of acute onset, life quality, lung function and TCM syndromes, so as to evaluate the efficacy and safety of Bushen Fangchuan Pian (Kidney-nourishing and Asthma-preventing Tablet) and Bushen Yiqi Fang (Kidney-nourishing and Qi-replenishing Prescription).

**2. Case selection**

**2.1 Research subject**

Modern medicine: COPD (disease-free period). TCM syndrome differentiation: Deficiency of kidney yang, deficiency of kidney qi, or deficiency of qi. Institutes in charge of the trial: Huashan Hospital Affiliated to Fudan University; Institutes participated: Xiyuan Hospital Affiliated to China Academy of Chinese Medical Sciences, Jiangsu Provincial Hospital of Traditional Chinese Medicine, Huaxi Hospital Affiliated to Sichuan University, and The First Affiliated Hospital of Henan College of Traditional Chinese Medicine. The cases are selected from out-patient department or in-patient department of different centers

**2.2 Diagnostic criteria**

**2.2.1 Diagnostic criteria of COPD**

Please take reference from the *Global Initiative for Chronic Objective Lung Disease (2008*) ^[1]^ and *Guidelines for Diagnosis and Treatment of Chronic Objective Lung Disease* issued in 2008 by Chinese Medical Association.

(1) Symptoms: Chronic cough, sputum production, short and difficult breath, wheezing, and chest distress, etc.

(2) Body signs: Over-distension of the chest, prolonged anteroposterior diameter, diminished cardiac dullness area, descent of liver and lung border, hyperresonant note on chest percussion , reduced breath sounds of the lungs, and expiratory flow retard, etc.

(3) Exclusion: Wheezing, breathlessness, chest distress and cough due to other diseases such as pulmonary interstitial fibrosis and pulmonary tuberculosis, which can be ruled out by chest film or CT.

(4) Examination of lung functions: After inhalation of bronchodilators, FEV1<80% estimated values and FEV1/FVC<70%. This may point to incompletely reversible airflow limitation.

Table 1: Symptoms of COPD

| Chronic cough | Beginning：intermittent; development: each day；deterioration: all day long（Cough only at night is rarely seen） |
| --- | --- |
| Chronic sputum production | Any forms |
| Difficult breath | Progressive, persistent, aggravation on exertion or respiratory infection |
| Exposure history to hazardous factors | Smoking, dust at work, chemical particles, smog from cooking or fuel |

**2.2.2 COPD stages**

It can be divided into two stages according to clinical symptoms:

(1) Diagnosis of COPD (acute exacerbation period) a. Apart from the symptoms such as breath difficulty, cough and/or sputum production, there are some acute changes, which necessitate modification of therapies. b. during the development, there are aggravated breathlessness and cough, combination of wheezing and chest distress, increase of sputum volume, change in color and viscosity of the sputum, and fever, etc.

(2) Diagnosis of COPD (stable period): The disease remains stable at a certain period, without dramatic changes to the symptoms such as cough, sputum production and short breath. No purulent sputum. Chest radiography: no exudative lesions in the lungs. Normal PBL count, and stable ventilation indexes of the lung.

**2.2.3 COPD Criteria for grading of severity**

Take reference from the *Global Initiative for Chronic Objective Lung Disease (2008*) ^[1]^ and *Guidelines for Diagnosis and Treatment of Chronic Objective Lung Disease* (2008)

The assessment of the severity of COPD should be performed according to the symptoms of the patients, the abnormality of the lung function, and complications (respiratory failure, heart failure), etc.

Table 2: Scaling of pulmonary function according to the severity of COPD

| Scale I  (Mild) | FEV1/FVC<70%,FEV1/ estimated value %≥80% | Mild obstruction of airflow, with or without cough and sputum production. At the time, the patient hasn’t yet realized the abnormality in lung function. |
| --- | --- | --- |
| Scale II  (Moderate) | FEV1/FVC<70%,50%≤FEV1/ estimated value %<80% | Further obstruction of airflow, aggravated symptoms with apparent short breath especially on exertion. At the time the patient will go to hospital because of dyspnea or aggravation of the disease. |
| Scale III  (Severe) | FEV1/FVC<70%,30%≤FEV1/ estimated value %<50% | Further aggravation of airflow, deterioration of short breath with recurrent acute onset. Thus the patient’s life quality will be affected. |
| Scale Ⅳ  (Extremely severe) | FEV1/FVC<70%,FEV1/ estimated value %<30%, or FEV1/ estimated value %<50%, combined by chronic respiratory failure | Severe obstruction of airflow, or combined by chronic respiratory failure. Thus the patient’s life quality can be significantly affected. If there is acute aggravation, his life will be endangered. |

**2.2.4 Evaluation of dyspnea**

It is evaluated with Dyspnea Measuring Scale of BMRC (Table 3)

Table 3: Scaling of functional dyspnea

| Scale 0 | No apparent dyspnea except there is violent activity |
| --- | --- |
| Scale 1 | Short breath when walking in hurry or climbing a duller slope |
| Scale 2 | Waking slower than the age cohorts because of dyspnea, or when walking at a normal pace, the patient needs to catch a breath. |
| Scale 3 | After waling for 100 meters or several minutes on the even ground, the patient should stop to catch a breath. |
| Scale 4 | Because of apparent dyspnea, the patient cannot leave the house or presents with short breath when taking off clothes. |

**2.2.5 Diagnostic criteria for** airway hyperreactivity

(1) Bronchial provocation test (if FEV_1_≥70%). Dilute the histamine with Sodium Chloride to different concentrations: 0.3%, 0.6%, 2.5% and 5%. Put them into 4 atomizers. Measure the patients’ pulmonary ventilation function after they have a rest. The indexes which should be measured: FVC, FEV1, PEF AND MMF. Each measurement should be conducted at lest twice. If the difference < 5%, the larger value of FEV1 is adopted; if the difference > 5%, the measurement should be performed again. Afterwards the patients breathe in atomized liquid of normal saline; the Mv of pulmonary function at this procedure is taken as the contrast value. And then the patients breathe in the histamine from low concentration to higher one. The pulmonary ventilation function is measured at fixed times. The judgment index for positive airway reactivity when inhaling histamine is: when the FEV1 decreases by 20%, the cumulant of histamine is less than 7.8μmol. The severity of reactivity can be divided into 4 scales according to histamine PD20 FEV1 (The cumulated histamine amount which decreases FEVI by 20%): <0.1μmol, Severe; 0.1～0.8μmol, Moderate; 0.9～3.2μmol, Mild; 3.3～7.8μmol, Extremely Mild; >7.8μmol, Normal.

(2) Bronchodilatation test (if FEV_1_<70%）. The basic FEV1 or PEF of the subjects should be measured first; at then they breathe in bronchodilators(200 ug～400 ug, salbutamol) with aerosol (MDI). After 15 min, the FEV1 and PEF are measured again. The improvement rate is then calculated. FEV1 or PEF improvement rate = [ (FEV1 or PEF after inhalation - FEV1 or PEF before inhalation) / FEV1 or PEF before inhalation] ×100 %。 If the improvement rate≥12%，and the absolute value of FEV_1_ improvement ≥200 ml, it is considered positive, indicating that there is airway hyperreactivity.

**2.2.6 TCM criteria for syndrome differentiation**

TCM criteria for syndrome differentiation: Take reference from the *Clinic Terminology of Traditional Chinese Medical Diagnosis and Treatment* issued by State Bureau of Quality and Technical Supervision. State criteria: GB/T16751.2-1997

(1) Deficiency of qi syndrome

Primary symptoms: Short breath, lassitude, deficient pulse/

Secondary symptoms: Spontaneous perspiration, listless, quietness, pale tongue.

(2) Deficiency of kidney syndrome

Primary symptoms: Short breath, aggravated on exertion.

Secondary symptoms: Tinnitus, weak loins and knees, hyposexuality, dizziness, forgetfulness, frequent urination, pale tone, sinking and thin pulse.

(3) Deficiency of kidney yang syndrome

Primary symptoms：Weak loins and knees, hyposexuality, fear of cold with cold limbs.

Secondary symptoms: Depression, frequent night urination, swelling lower limbs, breathless on exertion, withered hair and decayed teeth, pale tone, white fur, as well as sinking, tardy and feeble pulse.

(4) Deficiency of yin with abundant fire syndrome

Primary symptoms: Feverish sensation in palms, soles, and heart, hectic fever, night sweat.

Secondary symptoms: emaciation, dry mouth and throat, morbidly rosy cheeks, red or purple tongue, scanty fur or no fur, thin and rapid pulse.

If the patients present with two of the primary symptoms and at least two of the secondary symptoms, the diagnosis can be definitive.

**2.3 Inclusion criteria**

(1) Patients who are clinically diagnosed with chronic obstructive pulmonary diseases in stable stage. Severity scale: Ⅱ~Ⅲ;

(2) Patients who are diagnosed with deficiency of qi, kidney qi or kidney yang in TCM;

(3) Frequency of AECOPD in the past two years ≥2.5 times/year;

(4) 40 ≤ age ≤75, regardless of gender;

(5) Patients who haven’t presented with aggravation of COPD and taken glucocorticoid one month before selection.

(6) All the participants should take part in the trial voluntarily and sign the informed consent.

**2.4 Exclusion criteria**

(1) Combination by bronchiectasis, pulmonary tuberculosis, diffusing panbronchiolitis, pulmonary interstitial fibrosis, pulmonary infection, pneumothorax, pleural effusion, pulmonary embolism, and neuromuscular disorders which affect the breathing movements.

(2) Patients who are diagnosed with Deficiency of yin with abundant fire in TCM.

(3) Females who are pregnant or plan to be, and female who are in lactation.

(4) Patients with malignant tumor or blood diseases.

(5) Those who have been involved in other clinical trials in the previous three months.

(6) Those with severe damage to the heart, live and kidney (heart function scale 3~4, ALT and/or AST exceed the normal limit by more than 1.5 times, Cr surpasses the normal limit).

(7) Those who are accompanied by increase of airway reactivity.

(8) Other situations which the researchers believe to be inappropriate.

**2.5 Deletion criteria**

(1) After the trial, those who don’t meet the inclusion criteria or meet the exclusion criteria.

(2) After the trial, those who don’t take medicine according to rules.

(3) After the trial, those who haven’t any follow-up records.

(4) Those who have used a combination of drugs which affect the trial medication and the evaluation of efficacy and safety.

**2.6 Criteria and treatment for drop-outs**

(1) Patients who drop out voluntarily during the process of research.

(2) Those who drop out, fail to be followed-up or die due to various reasons unrelated to the disease.

(3) Those with incomplete data which might affect the judgment of efficacy and safety.

Treatment of drop-out cases: a. for the drop-out cases or those unable to follow-up, the researchers should take immediate measures to contact the patients by visit, telephone or mails, so as to record the last medication time and conduct the last measurement. b. For drop-out cases due to allergy, adverse effects, or invalid treatment, the researchers should take proactive treatment measures according to the individual situations. c. The trial conclusion and drop-out reason of all the drop-out cases should be written in case report.

**2.7 Trial termination criteria**

(1) Patients who are unable to receive medication due to adverse effects, complications or other usual physiological changes.

(2) Patients who abnormally break the blindness during the blind trial.

(3) Patients who present with apparent changes in TCM syndrome, which is unsuitable for the medication.

(4) During the process, there are serious mistakes in the therapies, thus unable to judge the efficacy.

(5) During the process, there are serious deviations, thus unable to judge the efficacy.

**3. Research method**

**3.1 Research design**

Adopt multi-center (five centers), randomized, double-blind, double simulation, parallel and controlled trial.

**3.2 Sample size**

The number of cases in the treatment group is the same with that of the control group. It is investigated that the effect of the treatment group is superior to that of the control group. The formula is made according to superiority clinical trial sample size. In view of the previous therapeutic effects and statistical requirement, the follow-up loss rate should be controlled below 15-20%. The cases for observation from the qualified ones required by the treatment group and the control group were both 120. There were 360 cases in total (include the drop-out cases). The total cases finally determined as well as the cases distributed to different centers are seen as follows in table 4.

**Table 4. Cases distributed to different centers**

|  | Huashan Hospital | Xiyuan Hospital Affiliated to China Academy of Chinese Medical Sciences | Huaxi Hospital Affiliated to Sichuan University | The First Affiliated Hospital of Henan College of Traditional Chinese Medicine | Jiangsu Provincial Hospital of Traditional Chinese Medicine | Total（cases） |
| --- | --- | --- | --- | --- | --- | --- |
| A group（cases） | 40 | 20 | 20 | 20 | 20 | 120 |
| B group（cases） | 40 | 20 | 20 | 20 | 20 | 120 |
| C group（cases） | 40 | 20 | 20 | 20 | 20 | 120 |
| Total（cases） | 120 | 60 | 60 | 60 | 60 | 360 |

**3.3 Randomized grouping**

The stratified randomization is used according to research centers and lung functions（50%≤FEV1/Estimated value %<80%, 30%≤FEV1/Estimated value %<50%）, so as to make sure the 360 cases can be distributed into the treatment group and control group at randomize. Each group includes 120 cases. All the cases should be told and treated by avoiding the influence of environment and other causative factors. `

Randomize the COPD patients into three groups: Group A, Group B, Group C.

Group A (control group): Bushen Yiqi Fang Simulation agent + Bushen Fangchuan Pian Simulation agent + Basic treatment (120 cases)

Group B (Bushen Yiqi Fang group): Bushen Yiqi Fang + Bushen Fangchuan Pian Simulation agent + Basic treatment (120 cases)

Group C (Bushen Fangchuan Pian group): Bushen Yiqi Fang Simulation agent + Bushen Fangchuan Pian + Basic treatment (120 cases)

Basic treatment: Treat the disease according to the GOLD scale （use Ventolin or bronchodilators if necessary）.

**3.4 Blind method**

**3.4.1 Blind design**

Double-grade blinding is adopted. The first grade is the treatment corresponding to the numbers (blind codes); the second grade is the code name corresponding to the three groups (randomized as A, B and C). The blind codes of the two grades are separately sealed up. Both are in duplicate and separately deposited in different clinical research institutes or sponsors. After collecting the cases, the database is established and the data are locked for double-grade unblinding. Before verifying the treatments corresponding to the code names, the code names of the groups must be statistically analyzed. If there are severe adverse events, death, or situations necessitate emergent treatment, it is up to the supervisor or chief researcher to decide whether to open the emergency letter or not. If the rate of blind code divulgement or reading of emergency letter exceeds 20%, the double blind trial will be considered invalid. Emergency letter: for every numbered trial drug, there is a corresponding emergency letter, which includes a slip of paper indicating the category of the drug. The letters are kept in the place of the main researchers of different institutes involved.

**3.4.2 Requirements for unblinding and emergent blindness-breaking**

(1) Requirements for unblinding

Double-grade unblinding is adopted. First grade unblinding: it is conducted before the data-analyzing. After the double input of all the CRF data into the computer and blind review, the data will be locked. Afterwards the personnel who keep the blind materials will reveal them for the first time. That is, to divide the groups corresponding to the case numbers into blind codes of two groups and tell the statisticians so as to statistically analyze all the data. Second grade unblinding: After the statistical analysis and completion of conclusion report, the second unblinding will be conducted at the summarization meeting for the clinical trial. And the treatment group and control group will be differentiated. Place of unblinding: The unit in charge of the clinical trial. Executive personnel: Chief researcher and statisticians of the unit in charge.

(2) Rules for emergent blindness-breaking

If there is severe adverse event, which impedes the progression of the trial and the selection of the treatment measures, emergent blindness-breaking can be carried. During the process, all the researcher, trial directors and clinical supervisors should take part in. The local administrative unit should be informed within 24 hours. The reason, time and place of blindness-breaking should be recorded in detail and signed. Afterwards the unit in charge and clinical supervisors should be timely informed. The case data should be kept intact.

**4. Treatment therapy**

**4.1 Preparation of trial medicines**

**4.1.1 Name, specification, usage and dosage of the medicines**

**(1)** Bushen Fangchuan Pian: Specification: 0.25g/tablet, produced by Shichuan Peiling Pharmaceutical Factory (State Drug Approval No. Z50020405 ); usage: 5 tablet/time, 3 times a day for oral administration

(2) Bushen Yiqi Fang: Each dose contains Huangqi, Yinyanghuo and Shengdihuang, etc. Diffuse the granular formulation of the medicine into hot water, and take it in the morning and evening, 125ml/time.

(3) Bushen Fangchuan Pian (Simulated Medicine): Specification is the same as the above-mentioned Bushen Fangchuan Pian, produced by Shichuan Peiling Pharmaceutical Factory; 5 tablet/time, 3 times a day for oral administration

(4) Bushen Yiqi Fang (Simulated Medicine): The form, character, flavor and usage are the same as Bushen Yiqi Fang.

(5) Salbutamol aerosol: Trade name: Ventolin, 100 ug*200 spray. Inhale the aerosol 100～200 ug each time (1~2 spray), and repeat it every 4 hours.

(6) Oxis Turbuhaler inhalant: Specification: 4.5ug*60 spray/bottle. Usage: Inhale the aerosol.

**4.1.2 Package of the medicines**

All the trial medicines are designed according to the subjects for visit medication. The tablets are all bottled. During each visit, the package contained Bushen Fangchuan Pian or placebo (drug A), Bushen Yiqi Fang or placebo (drug B) or placebos of both (drug C). Each subject is given a big kit which contains all the drugs needed in the experiment. Within it there is also a package in medium size containing the medicines for follow-up medication. Within the medium-size package, there are different small size packages containing the trial medicines.

**4.1.3 Preparation of the drug label**

Label: The form of the labels should be uniformed. Its contents: Batch number, serial number, name, function, indication, usage, storage and supply unit. It should also put on “Used only for clinical research”.

**4.2 Allocation of blind codes for the drugs**

An appointed person allocates the blind codes for the drugs. There are 360 codes in total, which are divided into three groups（Treatment group: 120; Control group: 120）. The whole process should be recorded in detail and follow certain rules required by the clinical drug coding. Besides the production of random numbers, for each case there is an emergency letter sealed in double-layer papers, indicating the drug code of patient. It also notifies the group which the case belongs to and the category and dosage of the drug. It is used for emergency unblinding. The letters and trial drugs will be sent to every research center all together and stored in the places of chief researchers.

**4.3 Allocation of drugs**

Allocate the drugs to the hospitals according to the randomly matched drug codes. All the centers select the patients for qualified ones. Distribute the drugs to the patients according to the order of visit. The amount of drugs should last exactly a course. And the Use of Trial Drugs Record List should be filled in timely by the administrator.

**4.4 Compliance**

Adopt drug counting method to monitor the subjects’ compliance. For each follow-up visit, the patient should take along the rest drugs so that the doctor can count and record them as well as calculate the compliance rate.

**4.5 Combination of drugs**

During the trial, all Chinese herbs or patent drugs with the function of nourishing the kidney and replenishing qi should be forbidden. If there are any complications, symptomatic treatments could be given according to the specific situations. If the patient has coronary artery disease, diabetes or hypertension, they should follow the direction to take their medicine. However, the name, factory, batch number, usage, dosage and administration time should be recorded in detail.

If there is acute exacerbation of COPD during the experiment, please follow the protocol for emergency treatment of AECOPD (see the attachment 1), and then continue the medication.

**4.6 Storage of drugs**

The trial drugs are stored by the different centers in clean, dry and airy rooms without direct sunshine. The quality of the drugs and its storage conditions should be checked periodically.

**4.7 Start-up stage**

Before allocating the patients to groups, there are two weeks of start-up stage. During this stage, the subjects inhale Salbutamol aerosol, 200ug/spray; if there are acute onset, the corresponding treatment can be carried out. If the conditions are stable after treatment, the patient can continue to receive the experiment.

**4.8 Disease course and follow-up visit**

Medication: 6 months; follow-up period: 6 months.

**5. Observation indexes and methods**

**5.1 Indexes for assessment of clinical therapeutic effects**

**5.1.1 Primary indexes for therapeutic effects**

(1) Pulmonary function

The lung vital capacity (VC) and FEV1 / estimated value %, FEV1/FVC％, and PEF are measured at 8~11 A.M. Before the test, the treatment drugs should be discontinued for more than 6 hours, violent activity should be discontinued for 2 hours, and patients should rest for 15 minutes.

(2) The time, frequency and severity of AECOPD^（2）^

The time, frequency and severity of AECOPD during the treatment stage and follow-up period are statistically analyzed.

Definition of AECOPD: The patient presents with two of the following symptoms (at least one primary symptom) which last at least 2 days. Primary symptoms: Dyspnea, profuse sputum, purulent sputum; secondary symptoms: Wheezing, pain in the throat, cough and common cold, etc.

Time of AECOPD: The duration from acute exacerbation to apparent improvement of the symptoms (the patient’s self-sensation).

Frequency of AECOPD: The interval between two episodes is at least a week, or they will be counted as one onset.

Severity of AECOPD: (1) Mild: no need for clinic service; oral intake of hormones, antibiotics and oxygen therapy are necessary. (2) Moderate: clinic service is necessary, and oral intake of hormones, antibiotics or oxygen therapy are required. (3) Severe: hospitalization or emergency treatment is needed.

(3) Evaluation of COPD patients’ life quality：ST. George’s respiration questionnaire (see the attachment) is used for evaluation of life quality.

**5.1.2 Secondary indexes for therapeutic effects**

(1) BODE index (see the attachment).

(2) Six minutes walking test (6MWT). The patient is asked to walk quickly for 6 minutes. During the period, his heart rate is observed. And the walking distances and dyspnea scales before and after the treatment are compared. The respiratory rate and heart rate before and after walking are calculated.

（3） Evaluation of inflammation indexes and HPA axis function

Draw 10 ml of blood and separate the serum (2-3ml) which is sent to Huashan Hospital for detection of TNF-α, etc. Draw 5 ml of whole blood and separate the blood plasma（1-2ml）to detect Hydrocortisone (see the attachment).

For typical case of deficiency of the kidney, the patient should receive cortisol circadian rhythm test, urinary-free cortisol test (Huashan Hospital). The former is tested at 8am, 4pm, 0am, and 8am (the next day).

（4）Quantitative scores of TCM syndrome (see the attachment)

(5) Psychological stress scale (see the attachment)-

**5.2 Evaluation of safety**

**5.2.1 General evaluation**

After the medication, the researchers should observe and record the possible discomforts of the patients during each treatment or follow-up visit. If there is any discomfort, the time of onset, severity, frequency, duration, measures and results should be asked. The researchers should also check the following indexes: heart rate, heart rhythm, blood pressure, routine blood test（red blood cell count, hemoglobin concentration, white cell count and blood platelets count, etc.）, routine urine test (test of white blood cell, red blood cell, protein and urine glucose), blood sugar, biochemistry (ALT, AST, TBI, BUN, Cr) and ECG.

**5.2.2 Adverse reactions**

(1)The severity of the adverse events can be classified into three degrees according to the subjective feeling of the subjects, whether the treatment progress is affected or not, and the harm to the subjects’ health: ①Mild adverse event: the patients present with bearable symptoms which don’t affect the treatment and the patients’ health and require no treatment. ②Moderate adverse event: the patients present with unbearable symptoms which affect normal life, harm the vital organs or systems and require discontinue of the medication or special treatment. ③ Severe adverse event: the patient present with severe symptoms which harm the vital organs or systems, shorten or endanger the patients’ life or lead to disability. In this case it requires discontinue of the medication and emergent treatment.

(2) The judgment of causality between adverse events and mediation

The judgment of causality between adverse events and mediation should take into consideration of the following aspects: ①Whether there is reasonable precedence relationship between the time of medication and the time when the adverse reaction appears; ② Whether or not the questionable adverse reaction belongs to the already known category; ③ Whether the adverse reaction can be explained by the patient’s pathological conditions, combination of drugs or therapies, and previous therapies; ④ Whether the questionable adverse reaction can be alleviated or disappear after discontinuation of medication or reduce the doses; ⑤ Whether the same reaction reappear after contacting the same drug again.

（3） The researchers should evaluate the possible causality between the adverse events and the trial drugs or combination of drugs according to the following six-scale classification criteria: ①Certain: there is a reasonable precedence relationship regarding the time of medication and the time when adverse event appear; the reaction disappears, alleviates or improves after discontinuation of drugs (according to the immune state of the body, some ADR reaction may appear several days after discontinuation); the reaction will reappear or become aggravated if the drug is used again; meanwhile there is evidence of documents; primary disease and other confounding factors are excluded. ②Probable: no history of repeated medication, the rest is the same with the above item, or although there is combination of drugs, the possibility of leading to adverse reactions can be basically ruled out. ③ Possible: There is close precedence relationship and document evidence; however, there is more than one drug which leads to adverse events, or there is progression of primary disease. ④Possibly unrelated: there is no close time correlation between the adverse reaction and medication, and no consistence between manifested reactions and the already known reactions of the drug; the progression of primary disease may also lead to such clinical manifestation. ⑤Undecided: it is hard to decide the causality and there is no document evidence. ⑥ Impossible: It is hard to determine the causality and there is no way to supplement the document.

（4）The treatment and report of adverse events

① Observation and record: The adverse events should be recorded honestly, and the patients should also tell their feelings honestly. The doctor should avoid inducible questions; at the same time of observing the therapeutic effects, the researchers should also pay close attention to the adverse events and unpredicted toxic or side effects (including symptoms, body signs and laboratory examinations). All the reactions should be recorded in detail, analyzed and concluded regardless of their correlation with trial drugs; besides, the occurrence rate of the adverse events should be calculated after observing, tracing and recording them. For all the adverse effects, the symptoms, severity, time of onset, duration, measures, process and turnover should be recorded; moreover, their correlation with the trial drugs should be evaluated, recorded, signed and dated.

②Treatment of adverse events: If there are adverse events, the main researchers will decide the treatment measures according to the conditions and determine whether to terminate the observation. For mild adverse events, if they do not harm the patients’ health, symptomatic treatment can be used and there is no need for special treatment. For moderate adverse events, the trial should be discontinued and special treatment should be given. For severe adverse events which may endanger life, the clinical trial should be stopped immediately and emergent treatment should be carried out according to the contingency plans (e.g., anaphylactic response, heart failure, respiration failure and pneumothorax) made by the participating institutes. Meanwhile the treatment results should be recorded by the clinical supervisor and reported to the Ethics Committee of the hospital. If the adverse events still linger without alleviation, follow-up visits should be conducted until the recovery or stabilization. The researchers should trace and investigate the cases of drug discontinuation due to adverse reactions, and record in detail the process and result of treatment.

③ Report: If there is severe adverse event during the trial, it should be reported to the Ethics Committee, Research Department, project director and provincial or state FDA. The SFDA should be informed within 24 hours.

**6. Clinical observation and follow-up steps**

**6.1 The first visit (-W2)**

(1) Determine the treatment;

(2) Obtain the case history and demographical data;

(3) Conduct comprehensive body examinations, including examination of body temperature, pulse, respiration, blood pressure, body weight, height, tongue manifestation, and pulse manifestation, etc.;

(4) Record the clinical symptoms as well as combined drugs and dose in detail; evaluate the severity of COPD, and begin the start-up period;

(5) Educate the COPD patients (See the attachment 7);

(6) Tell the patients the time of next follow-up visit.

**6.2 The first interview (baseline) (M1)**

(1) Conduct comprehensive body examinations, including examination of body temperature, pulse, respiration, blood pressure, etc.;

(2) Routine laboratory examinations, including routine blood and urine tests, liver and kidney functions, blood sugar and electrocardiography; draw 10 ml of blood and separate the serum (2-3ml), and draw 5 ml of whole blood and separate the blood plasma（1-2ml）, stored in cryogenic box; x-ray film and lung functions;

(3) Fill out the St George’s respiration questionnaire, BODE indexes, 6 MWD, quantitative scores of TCM syndrome, metal stress scale, etc.

(4) Evaluate the severity of COPD and determine the basic medication according to the conditions of disease.

(5) Record the clinical symptoms as well as combined drugs and dose in detail.

(6) Distribute the trial drugs according to the sequence of selection and record the corresponding drug codes in CRF which remain unchanged throughout the research; fill out the drug release report.

(7) Distribute the COPD journals and inform the patients of the next visit time.

**6.3 The second interview (M2)**

(1) Retrieve the COPD journals and drugs, evaluate the medication compliance, and dismiss the unqualified.

(2) Fill out the St George’s respiration questionnaire and quantitative scores of TCM syndrome;

(3) Conduct comprehensive scoring of the symptoms and body examinations, including examination of body temperature, pulse, respiration, blood pressure, etc.;

(4) Record the frequency, time and severity of AECOPD

(5) Distribute the trial drugs and fill out the drug release report.

a. Recorded the combination of drugs and doses;

b. Record the adverse events and their corresponding treatment;

c. Distribute the COPD journals and inform the patients of the next visit time.

**6.4 The third interview (M4)**

(1) Retrieve the COPD journals and drugs, evaluate the medication compliance, and dismiss the unqualified.

(2) Fill out the St George’s respiration questionnaire and quantitative scores of TCM syndrome;

(3) Conduct comprehensive scoring of the symptoms and body examinations, including examination of body temperature, pulse, respiration, blood pressure, etc.; recheck the routine blood and urine tests, liver and kidney functions, blood sugar and electrocardiogram;

(4) Record the frequency, time and severity of AECOPD

(5) Distribute the trial drugs and fill out the drug release report.

a. Recorded the combination of drugs and doses;

b. Record the adverse events and their corresponding treatment;

c. Distribute the COPD journals and inform the patients of the next visit time.

**6.5 The forth interview (M7)**

(1) Retrieve the COPD journals and drugs, evaluate the medication compliance, and dismiss the unqualified.

(2) Conduct comprehensive scoring of the symptoms and body examinations, including examination of body temperature, pulse, respiration, blood pressure, etc.;

(3) Fill out the quantitative scores of TCM syndrome, St George’s respiration questionnaire, BODE indexes, 6 MWD, metal stress scale, etc.

(4) Record the frequency, time and severity of AECOPD.

(5) Routine laboratory examinations: recheck the routine blood and urine tests, liver and kidney functions, blood sugar and electrocardiography; draw 10 ml of blood and separate the serum (2-3ml), and draw 5 ml of blood and separate the blood plasma（1-2ml）, stored in cryogenic box; Lung functions;

(6) Record the combination of drugs and doses in detail as well as adverse events and their corresponding treatment.

**6.6 The fifth, sixth interviews (M10, M13)**

Conduct comprehensive scoring of the symptoms and body examinations, including examination of body temperature, pulse, respiration, blood pressure, etc.; Record the frequency, time and severity of AECOPD; Record the combination of drugs and doses in detail.

**7. Criteria for evaluation of therapeutic effect**

7.1 Criteria for evaluation of therapeutic effect with modern medical indexes

The frequency, time and severity of AECOPD, evaluation of COPD patients’ life quality, lung functions, BODE indexes, 6MWT, peripheral blood inflammation indexes, and quantitative scores of TCM syndrome and evaluation of HPA axis function; All of these indexes are statistically analyzed to compare the inter-group and intra-group differences before and after the treatment.

7.2 TCM criteria for evaluation of therapeutic effect

Take reference from P*rinciple of Clinical Study on New Drug of Traditional Chinese Medicine*.

(1) Clinically controlled: Clinical symptoms and body signs disappear or basically disappear; treatment index ≥95%.

（2）Markedly Effective: Clinical symptoms and body signs improve significantly; treatment index ≥65%.

（3）Effective: Clinical symptoms and body signs improve; treatment index ≥30%.

（4）Non-effective: Clinical symptoms and body signs do not improve significantly or even aggravate; treatment index ≤30%.

The treatment index (n) is calculated according to the formula: n=（scores before the treatment-scores after the treatment）/scores before the treatment×100%。

**8 Contents of statistical analysis**

8.1 Concepts

(1)Safety set (SS): Include all populations after randomization and at least one time of medication as well as a post-treatment safety evaluation.

(2) Full analysis set (FAS): Include all populations after randomization and at least one time of medication as well as at least one post-treatment measurement of main index.

(3) Per protocol set (PPS): Include those who are consistent with the following conditions: effective baseline value; conform to the protocol and the inclusion criteria; complete all the evaluations; good compliance (80%-100%).

8.2 Analysis of completion situation

Analyze cases of drop-out and discontinuation and patients’ compliance. Statistical method for numeration data is used.

8.3 Analysis index of baseline balance

(1) Measurement data: Age, disease course, average occurrence in the past two years, indexes of lung function. Statistical method for measurement data is used.

(2) Numeration data: Sex, smoking condition, complication, etc. Statistical method for numeration data is used.

8.4 Indexes for analysis of therapeutic effect

(1) Lung function indexes: Include FEV_1_/Estimated value％, FEV_1_/FVC％ and PEF. Statistical method for measurement data is used.

(2) The frequency, time and severity of acute onset；Statistical method for measurement data is used.

（3）St George’s respiration questionnaire, psychological stress scale, BODE indexes, 6MWT, peripheral blood inflammation indexes, quantitative scores of TCM syndrome and HPA axis function; Statistical method for measurement data is used.

(4) Indexes for safety analysis

Laboratory indexes: Routine urine and blood tests; liver (ALT, AST and TBIL), renal function examination (BUN, Cr), blood sugar, electrocardiography. Statistical method for measurement data is used to analyze WBC, RBC, HBC, NE, BUN, Cr, AST and ALT, ETC. Other indexes and the adverse events are analyzed by statistical method for the numeration data.

8.5 Statistical analysis method

SPSS11.5 statistical software is used for analysis. The measurement data is indicated by mean ± standard deviation. For the data with normal distribution, paired *t* test is used. The inter-group comparison is conducted with one-factor analysis of variance. For the data without normal distribution, rank sum test is used for inter-group comparison andχ^2^ test is used for group rate comparison. P <0.05 is considered statistically significant.

**9 Management of the data**

9.1 The writing and transfer of case report

The case report is written by the doctor who has participated in the trial. Every case selected should have a complete case report. The case report, once completed, should be checked by the supervisor; afterwards it is transferred to the data administrator for data entry and management.

9.2 The design and establishment of database

Record all the information in CRF table with a specialized clinical experimental database designed by Chinese academy of Traditional Chinese medicine. The format of the database should be close to that of the CRF table so as to facilitate the data entry.

9.3 Data coding

Encode the variables in the CRF table and keep the codes unchanged during the whole process of clinical research.

9.4 Data entry

Enter the CRF data by highly trained specialists from the research centers.

9.5 The audit of data

The audit of data can be divided into two forms: artificial audit and system audit. The former refers that the administrator checks the consistency and logic of the data so as to find the mistakes and produce the question list. SAS software sets the limit of all variables, and rules out automatically the unqualified data by running the system program. The question list is sent to the clinical supervisor who transfers it to the researcher for reconfirmation. The related revise should be signed and dated by the researcher.

9.6 data locking

The researcher revises the data for the last time after the return of all question lists. All the corrections and updates should be recorded and filed. After verifying the data, the data administration meeting will be opened so that the corrections and updates can summarized. At last the data administrator announces the locking of data and keeps the cipher code. The statistical analysis prospectus will not be changed after the data lock. The data will be transferred to the statistics department for analysis.

9.7 Data storage

All the data should be kept according to the requirements of GCP. After the experiment, all the original copy of case reports and records for use of clinical drugs should be checked, signed and stamped by the supervisors, head researchers and clinical base, and finally sent to the unit in charge. The unit in charge will establish the database and process the data. Statisticians will analyze the data and material of the participating units, and the unit in charge will complete the summary of the clinical trial.

**10 Quality control and guarantee of the clinical trial**

10.1 Measures for quality control

10.1.1 Quality control in the laboratory

All the participating units should conduct tests according to standard operating procedures and quality control procedures, and provide range of normal values for laboratory examination. If there are any changes, it should be timely explained.

10.1.2 Qualification inspection of researchers

The researchers should possess expertise knowledge, qualification and ability for clinical experiments, and be investigated and relatively immobile.

10.1.3 Training before the clinical trial

Before the trial, the researchers are trained to fully understand the trial schedules and the index connotations. The description of subjective symptoms should be objective and the question should not be inductive. For the objective index, it should be examined by regulated time and method. The adverse reactions and unpredicted side or toxic effects should be observed and traced.

.10.1.4 Compliance

Inquire the medication compliance of subjects by drug counting method and strengthen the follow-up effect so as to guarantee the subject’s compliance.

10.1.5 Adverse reactions

Inform the subjects of possible adverse reactions of the drugs and the corresponding measures.

10.2 Measures of quality control

Establish multi-center trial coordination committee; Huashan Hospital Affiliated to Fudan University takes charge of the committee, and the main researchers of the participating units serve as the members. The committee is responsible for the implementation of the whole experiment and irons out problems during the process of trial.

The head researcher should strengthen quality surveillance of the clinical trial in his own center.

**11. Predicted progress and completion time**

11.1 Start-up time of the clinical trial

The clinical trial begins when the protocol is approved by Ethics Committee and drugs, research materials and fund are ready.

11.2 Mid-term coordination meeting

Whether the meeting is opened or not relies on the progression of the trial.

11.3 The completion time of the trial

Twenty-four months after the initiation.

11.4 The time of collecting, analyzing and summarizing of the trial data

The statistical analysis and summary should be completed within 3 months after the trial completion and the data lock.

Attachment

**1 Emergency treatment plan of AECOPD**

The patients who with AECOPD are treated according to the treatment guide to this disease. Please refer to the *Guidelines for Diagnosis and Treatment of Chronic Objective Lung Disease* issued in 2008 by Chinese Medical Association.

**2. ST. George’s respiration questionnaire; please refer to COPD journals**

**3 BODE indexes and evaluation**

BODE indexes include such indexes of body weight, airway obstruction, dyspnea and exercise capacity. It is named after the 4 physiological parameters needed to be measured: B refers to body weight indexes, O refers to the degree of airflow obstruction, D refers to dyspnea, and E refers to exercise capacity. It is Celli, from the Sr.Elizabeth medical center Affiliated to Tufts Medical College, who invented the BODE indexes. It only takes a few minutes (0~10 minutes) to measure the indexes. Researches indicated that for each increase of 1 score, the death risk enhances by 33%.

**Table 5. BODE indexes and evaluation**

| Items | 0 score | 1 score | 2 scores | 3 scores |
| --- | --- | --- | --- | --- |
| B | >21 | ≤21 | － | － |
| O | FEV1%/ Estimated value ≥65% | 50%≤FEV1% Estimated value ≤64% | 36%≤FEV1% Estimated value≤49% | FEV1% Estimated value≤35% |
| D* | Scale 0-1 | Scale 2 | Scale 3 | Scale 4 |
| E | ≥350 meters | 250～349 meters | 150～249 meters | ≤150 meters |

*Note: for the objective evaluation of dyspnea, we can classify the functional dyspnea of COPD into 5 scales: Scale 0, the patient doesn’t present with dyspnea if violent activities are absent; scale 1, the patient presents with short breath when walking in hurry or climbing a duller slope; scale 2,waking slower than the age cohorts because of dyspnea, or when walking at a normal pace, the patient needs to catch a breath; scale 3: After waling for 100 meters or several minutes on the even ground, the patient should stop to catch a breath. Scale 4, because of apparent dyspnea, the patient cannot leave the house or presents with short breath when taking off clothes.

The scores of each item should be evaluated separately and the total scores are summarized by adding them up.

**4. Quantitative scores of TCM syndrome**

Please take reference from P*rinciple of Clinical Study on New Drug of Traditional Chinese Medicine*. The treatment index (n) is calculated according to the formula: n=（scores before the treatment-scores after the treatment）/scores before the treatment×100%。

**Table 6 Quantitative scores of TCM syndrome**

| Symptoms | | Normal | Mild | Moderate | Severe | Scores |
| --- | --- | --- | --- | --- | --- | --- |
| Main symptoms | Weak loins and knees | No | Relatively mild | Occasionally | Frequently |  |
|  | Short breath | No | Mild short breath without affection on normal life and work | Short breath with affection on normal life and work | Short breath even when the patient is at ease with significant affection on normal life and work |  |
|  | Fear of cold with cold limbs | No | Mild fear of cold | Fear of cold with cold limbs | Severe fear of cold with cold body which cannot be relieved by warmth |  |
|  | Listlessness | No | Mild listlessness | Listlessness with energy barely enough to maintain daily work | Severe listlessness with inability to maintain daily work |  |
|  | Lassitude | No | Lassitude on exertion | Lassitude on movement | Lassitude even at ease |  |
| Secondary symptoms | Frequent night urination | No | Twice night urination | Three times of night urination | More than four times of night urination |  |
|  | Spontaneous perspiration | No | Slightly moist skin | Moist skin | Perspiration |  |
|  | Dizziness and forgetfulness | No | Occasional dizziness and forgetfulness | Frequently | Appear every day |  |
|  | Quietness | No | Seldom speak | Unwilling to speak | Reluctant to speak |  |
|  | Swelling lower limbs | No | Slightly depressed when pressed | Depressed when pressed | Deeply depressed when pressed |  |
|  | Wither the hair and decayed teeth | No | Listless hair with unstable teeth | Withered hair with unstable teeth | Withered hair with loss of teeth |  |
|  | Tinnitus | No | Occasional tinnitus | Frequent tinnitus | Refractory, unstopped tinnitus |  |
| Total scores | |  | | | |  |

Tongue manifestation: it is described in specific words without scoring.

Main symptoms are scored 2, 4 and 6 according to their severity, while secondary symptoms are scored 1,2 and 3 according to severity . No 0 score. Total scores＝scores of the main symptoms＋scores of the secondary symptoms.

Meanwhile each item is scored separately.

**5. Standard operating procedures of 6MWT**

5.1 Indications and cautions

The optimal indication is moderate or severe diseases of the lung and heart under medical intervention.

5.1.1 Evaluate the therapeutic effect of patients with moderate or severe diseases of the lung and heart: lung transplantation, pulmonary resection, pneumoresection, lung recruitment, COPD, pulmonary artery hypertension, and heart failure.

5.1.2 Measure the functional state of the patients: COPD, pulmonary fibrosis, heart failure, peripheral angiopathy, fibromyalgia syndrome and senile patients.

5.1.3 Predict the occurrence rate and death rate: heart failure, COPD and idiopathic pulmonary hypertension.

5.2 Contraindications

5.2.1 Absolute contradictions: Early stage of instable angina pectoris and heart infarction.

5.2.2 Relative contradictions: Persistent heart rate ＞120 times, systolic pressure ＞180mmHg and diastolic pressure ＞100mmHg.

5.3 Safety measures and preparation

5.3.2 Proper location of the test should be selected to ensure immediate, correct treatment of emergencies. The park location of the ambulance should be decided by the doctor.

5.3.2 Oxygen, Nitroglycerin for sublingual administration, aspirin, and aerolin (metered-dose inhaler or spray gun) should be prepared.

5.3.3 The technicians should have the skill of cardio-pulmonary resuscitation

5.3.4 Physicians should be present throughout the test.

5.3.5 Chronic oxygen therapy should be conducted according to the standard oxygen flow or verbal order of the physician.

Situations necessitate immediate discontinuation of 6MWT: a. chest pain; b. unbearable dyspnea; c. lower limb spasm; shaking, staggering walk; d. profuse perspiration; e. pale or dark complexion.

If the test is discontinued due to the above-mentioned situations, the patient should sit down or lie on the back according to the severity of symptoms or the judgment of the technician. The measurement of blood pressure, pulse and saturation of blood oxygen, as well as the evaluation of the physician, should be conducted immediately. Oxygen therapy is given whenever necessary.

5.4 Requirements of the test location

The test should be conducted indoors. The place should be a long, even, closed, hard and quiet corridor 30 meters in length. The corridor should be marked at every 3 meters and the turning point is marked with a circular cone (such as the reddish yellow circular cone for transportation). There are a starting point and an ending one, which are marked with bright ribbons.

5.5 Necessary equipment

5.5.1 Time keeper (or stopwatch)

5.5.2 Two small circular cones to mark the turning point

5.5.3 A movable chair

5.5.4 A Work table clipped on the clipboard

5.5.5 Oxygen supply equipment

5.5.6 Blood pressure measuring instrument

5.5.7 Telephone

5.5.8 Automatic electronic defibrillator

5.6 Preparation work of the patients

5.6.1 The patients should put on comfortable clothes

5.6.2 The patients should wear comfortable shoes

5.6.3 The patients should be assisted by their daily mobility aids (a cane or walker, etc.)

5.6.4 The patients should proceed with their daily medical treatment.

5.6.5 The patients should be allowed to take small amounts of food in the morning or evening before the test.

5.6.6 The patients should not be allowed to take violent activities within 2 hours before the test.

5.7.1 The test should be repeated the day when it is conducted so as to reduce to aberration rate.

5.7.2 Before the test, the patient should avoid activities that may make him excited.

5.7.3 Before the test, the patient sits at the place close to the starting point for more than 10 minutes. During the time, the doctor examines the blood pressure, pulse and that if there are contraindications or not and whether or not the clothes and shoes are comfortable. After all of these, the first part of the work table is completed.

5.7.4 Pulse hemoxometer is an applicable device; it is used to measure and record the baseline pulse rate and SPO2. The operator should amplify the signal to the maximum and reduce the influence of movements according to the instruction. Meanwhile, he should check that whether the data recorded are stable or not, and record the quality of the pulse rate and signal at regular times.

5.7.5 The doctor tells the patient to stand up, and measures his baseline dyspnea and overall fatigue with Borg measurement method. （Table2 is an illustration of Borg method）

5.7.6 The doctor makes sure that all the equipment is ready (time keeper, clipboard, Borg standards, and work table) and transfers them to starting point.

5.7.7 The doctor should inform the patient of the following information: “you should walk along the long corridor back and forth within 6 minutes, which is a long time. So, you should make you best. You may feel hard to breathe and tired, so you can slow down, stop or even have a rest by leaning against the wall when permitted. However, once you regain you strength, please keep walking.”

“You will turn back quickly at the circular cone; now I will give you a demonstration, please pay attention.”

“Are you ready? I will count you steps.”

“Please remember, you should walk as quickly as possible for 6 minutes. Do not run or walk slowly. ”

Now you can begin or begin at any time after preparation.

The Brog measurement method should be printed on a piece of paper and put at an eye-catching place. It will be shown to the patient who shall be asked, “Please grade you dyspnea with this criteria, and point out you scale.” After the test, the patient is reminded of his pretest level and asked to re-grade his dyspnea.

5.7.8 The patient stands at the starting point, so does the doctor. The doctor stays at the starting point while the patient begins to walk. The time is kept immediately.

5.7.9 Everyone should be silent except the doctor, who encourages the patient in standard Mandarin. Do not interfere with the patient or omit the walking data. Each time the patient returns to the starting point, the doctor should make a mark on the work table. This gesture is supposed to be seen by the patient, and you can use exaggerative body language to press the time keeper, like using a stop watch during the match,

One minutes after, the patient is told, “Well done, there is still five minutes left.”

When there is still 4 minutes, you should tell the patient, “Keep walking, and there is still 4 minutes left”

When there is still 3 minutes, you should tell the patient, “Well done, and you have covered half of the way.”

When there is still 2 minutes, you should tell the patient, “Keep walking, and there is only 2 minutes left.”

When there is still 1 minute, you should tell the patient, “Well done, and there is only 1 minute left.”

If the patient stops and needs a rest during the test, you can tell the patient: “You can lean against the wall to have a rest if you would like, and when you regain your strength, please go on.” At this time, do not stop the time keeper. If the patient stops and refuse to walk again before it is completed (or you decide that the patient’s conditions do not allow the continuation of walking), he should sit in a wheelchair and have a rest. The walking distance, ending time and reason for the pre-termination should be recorded on the work table.

When there is still 15 seconds left, please tell the patient, “It is about to end, and when time comes, please stop immediately at the place where you stand. And I will come to you at once.”

When the time keeper rings or vibrates, please tell the patient to stop, and come to him. If the patient is very tired, give him a chair to sit on, and mark the place where the patient stops with a ribbon.

5.7.10 Measurement after the test: Record the degree of dyspnea and fatigue with Borg method, and ask: “what do you think enables you to walk so long.”

5.7.11 If the pulse hemoxometer is used, please measure the SPO2 and pulse rate immediately. Afterwards remove this equipment.

5.7.12 Please record the steps which the patient takes on the worktable

5.7.13 Use the marks on the wall and record the last extra walking distance (the value of walking distance after the last circle). Calculate all the walking distances and change them to the nearest whole numbers, which are recorded on the work table.

5.7.14 Congratulate the patient for his effort and offer him a glass of water.

**6. The process and collection of blood samples**

6.1 Separation of 2~3 ml of serum from 10 ml of blood

Extract 10 ml of blood with common tube, and put it at room temperature (22～25ºC) for 30~60 minutes for spontaneous agglutination. The tube must be corked and vertically placed, with the opening upward. Hence, there is little chance for vibration and the clotting process will be accelerated. Within 2 hours it is centrifugalized at the centrifugal machine. Setting: whole blood 1500～2000r/min for 10~15 minutes of centrifugalization. If there is no centrifugal machine, the whole blood should be kept at 4～8 ºC for 24 hours. After the complete separation of serum, remove the supernatant with a pipette to a sterile serum tube.

6.2 Separation of 1~2 ml of plasma from 5 ml of whole blood

Add the decoagulant and aprotinin (for concentration and amount, please refer to the note) to the blood-collection tube, which is placed at 4℃ for precooling, and then collect 1.5 ml of whole blood accurately. Mix the contents in the tube slowly and afterwards centrifugalize the mixture at a low temperature (4℃, 4000r/min, 15-20 min). Collect 0.5 ml of plasma and keep it at a low temperature (-80℃). If the collected blood can not be centrifugalized immediately, it should be stored in 4℃ freezer for less than an hour.

Note: The concentration and amount of decoagulant and aprotinin

(1) Decoagulant: 0.3MEDTA.2Na concentration (**20ul/ml**) or 1% heparin (**10ul/ml**);

(2) Aprotinin （500 IU / ml ）. There are two kinds of aprotinin: liquid (the concentration will be noted on the label) and solid (10800IU/mg). The solid form of aprotinin can be dissolved in normal saline, so its concentration can be adjusted to 500IU/20 ul.

6.3 Requirements for sample storage

The samples should be kept immediately in -80° freezer. Throughout the transportation, the samples can not be taken out. In Huashan Hospital, all of the samples are detected. The samples should be labeled with case codes and collection date. Blood serum should be kept in dry ice for transportation.

**7. Education for patients with COPD**

COPD, a preventable and treatable disease, has apparent extrapulmonary effects. Its severity varies from individual to individual. The pathological change is characterized by irreversible obstruction of airflow, which is generally progressive and related to abnormal inflammatory reactions of the lung due to toxic particles or gases.

7.1 Risk factors

The risk of COPD is related to the overall load of particles inhaled in one’s life.

Smoking, the commonest risk factor at any place in the world, includes the use of tobaccos, pipes, cigars and other popular smoking products as well as environment tobacco smoking (ETS).

Occupation dust and chemicals (steam, stimulant and smog) are also risk factors when people are exposed to them for too long.

Indoor air pollution due to cooking or biofuel in a room with poor ventilation may also be a risk factor for women in developing countries.

Outdoor air pollution is less harmful, although it may increase the overall load of inhaled particles.

Besides, during the embryonic stage and childhood, any factor which may affect the lung development (such as low birth weight and respiratory infection, can increase the risk of COPD.

Prohibition of smoking: Promote the dissemination of smoke abatement information with persistent effort, encourage the establishment of policies and plans for widespread tobacco control, set up no-smoking schools, public facilities and work places, and persuade the patients to build smoke-free homes.

Occupation exposure: Stress primary prophylaxis by decreasing or eliminating the exposure to occupation particles or gases. Secondary prophylaxis is also important by monitoring or early monitoring.

Indoor and outdoor pollution: Adopt measures to reduce or avoid air pollution due to biofuel, cooking or heating fuel. Advise the patient to pay attention to air report, so as to decide whether to go outdoors or stay indoors.

7.2 Family management of acute exacerbation

Bronchodilators: Increase the dose and/or application frequency of the currently used bronchodilators. Β2-excitomotor is the first choice. If the anticholinergic agents are not used, the above-mentioned drugs can be added till the symptoms improve.

Glucocorticoids: If baseline FEV1<50% Estimated value, it is advisable to add oral Prednisolone (30～40mg/d, 7～10d in total) on the basis of bronchodilators. If the acute exacerbation is not accompanied by acidosis, the oral intake of Prednisolone can be replaced by aerosol inhalation of Budesonide

7.3 Rehabilitation plans

The objective of rehabilitation is to improve the symptoms, life quality, and ability of carrying out daily activities. It mainly includes the following aspects:

Exercises

Nutrition consultation

Education

Patients with COPD in different degrees can all benefit from exercises which can improve the exercise tolerance, dyspnea and fatigue. The effect can last even after the rehabilitation. An effective rehabilitation program will last at least 6 weeks. The longer the time, the better the effect. After rehabilitation, the effect will gradually wane. However, as long as the patient continue exercises at home, his or her health state will be better than that before the rehabilitation.

7.4 Oxygen therapy

Long term oxygen therapy (>15h/d) can improve the survival rate of patients with COPD, with favorable influence on pulmonary hemodynamics, hematologic change, motor ability, pulmonary mechanical force and mental state.

The objective of long-term oxygen therapy is to improve basic resting PaO_2_ (sea level) to 810kPa (60mmHg), or improve SaO_2_ to 90%, thus ensuring adequate oxygen delivery for maintenance of vital organ functions.

**8．References**

1. GOLD Committees. Global initiative for chronic obstructive lung disease. Global strategy for the diagnosis, management, and prevention of chronic obstructive pulmonary disease (updated 2008). Medical Communications Resources, Inc, 2008.

2. ZHOU Yu-min, WANG Xiao-ping, and ZENG Xiang-yi, et al. Randomized Double-blind Parallel Controlled Study of Theophylline in Treating COPD. Chinese Journal of Tuberculosis and Respiratory Diseases; 2006(9); 29:577-582.
