# Supplementary figures and images for: Effects of Two Chinese Herbal Formulae for the Treatment of Moderate to Severe Stable Chronic Obstructive Pulmonary Disease: A Multicenter, Double-Blind, Randomized Controlled Trial
Source: PLoS One. 2014 Aug 13;9(8):e103168. doi: 10.1371/journal.pone.0103168 (PMC4132093; doi:10.1371/journal.pone.0103168)

**Ethics approval document**


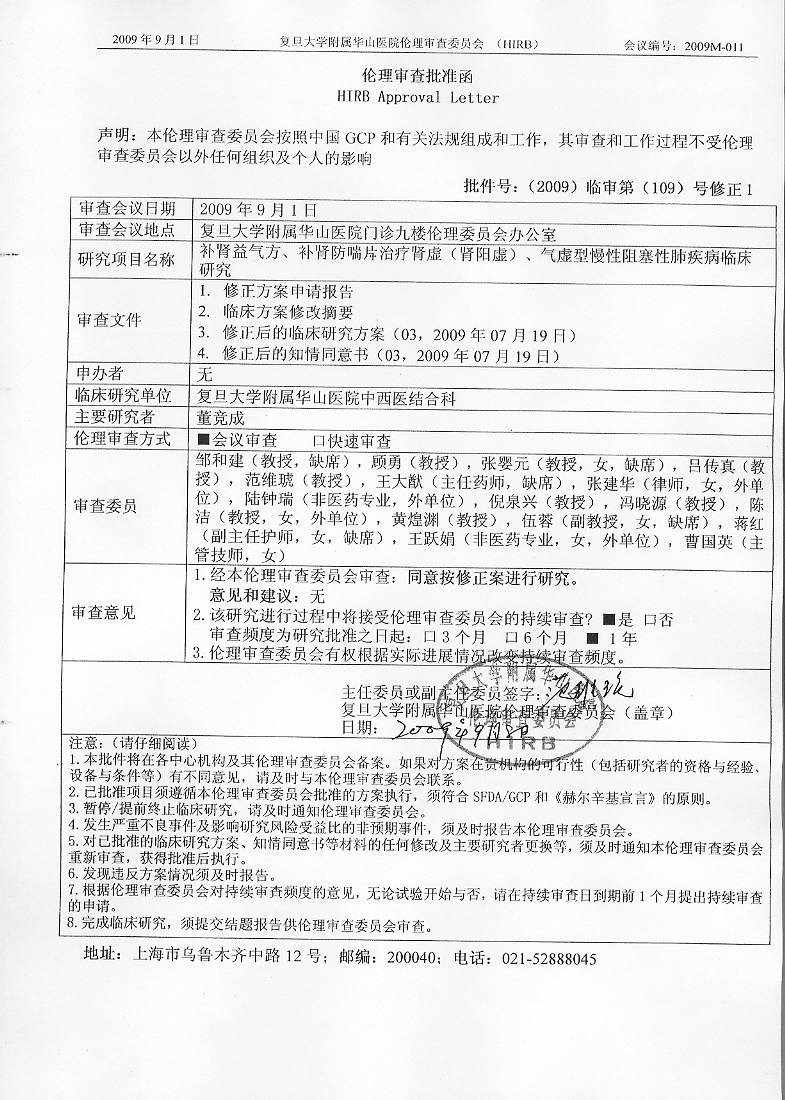

Supplement: Ethics Approval Document S1 — Ethics approval document. (DOC) [file pone.0103168.s003.doc]
